# Plant HP1 protein ADCP1 links multivalent H3K9 methylation readout to heterochromatin formation

**DOI:** 10.1038/s41422-018-0104-9

**Published:** 2018-11-13

**Authors:** Shuai Zhao, Lingling Cheng, Yifei Gao, Baichao Zhang, Xiangdong Zheng, Liang Wang, Pilong Li, Qianwen Sun, Haitao Li

**Affiliations:** 1 MOE Key Laboratory of Protein Sciences, Beijing Advanced Innovation Center for Structural Biology, Tsinghua-Peking Joint Center for Life Sciences, School of Life Sciences, Tsinghua University, Beijing, 100084 China; 2 Department of Basic Medical Sciences, School of Medicine, Tsinghua University, Beijing, 100084 China

**Keywords:** X-ray crystallography, Plant molecular biology

## Abstract

Heterochromatin Protein 1 (HP1) recognizes histone H3 lysine 9 methylation (H3K9me) through its conserved chromodomain and maintains heterochromatin from fission yeast to mammals. However, in *Arabidopsis*, Like Heterochromatin Protein 1 (LHP1) recognizes and colocalizes genome-wide with H3K27me3, and is the functional homolog of Polycomb protein. This raises the question whether genuine HP1 homologs exist in plants. Here, we report on the discovery of ADCP1, a plant-specific triple tandem Agenet protein, as a multivalent H3K9me reader in *Arabidopsis*, and establish that ADCP1 is essential for heterochromatin formation and transposon silencing through modulating H3K9 and DNA methylation levels. Structural studies revealed the molecular basis underlying H3K9me-specific recognition by tandem Agenet of ADCP1. Similar to human HP1α and fly HP1a, ADCP1 mediates heterochromatin phase separation. Our results demonstrate that despite its distinct domain compositions, ADCP1 convergently evolves as an HP1-equivalent protein in plants to regulate heterochromatin formation.

## INTRODUNCTION

Heterochromatin mediates various functions such as transposon silencing and nuclear organization.^1,2^ The formation and functional maintenance of heterochromatin require Heterochromatin Protein 1 (HP1).^3^ HP1 recognizes histone H3 lysine 9 trimethylation (H3K9me3) through its chromodomain and this interaction can be switched off by phosphorylation at adjacent H3 serine 10 (H3S10ph).^4–7^ The chromo shadow domain of HP1 mediates dimerization of HP1 and its association with a repertoire of proteins.^8^ HP1 was also reported to mediate heterochromatin phase separation in flies and humans.^9,10^ As an H3K9me3 reader, HP1 is conserved from fission yeast to mammals. However, in *Arabidopsis*, Like Heterochromatin Protein 1 (LHP1) recognizes and colocalizes genome-wide with H3K27me3, and is the functional homolog of Polycomb protein.^11,12^ This raises the question whether genuine HP1 homologs exist in plants.

HP1 belongs to the “Royal Family” proteins, which contain Chromo, Tudor, PWWP, MBT and Agenet domains.^13^ Agenet domains are the plant-abundant “Royal Family” members and conserved among species. Several Agenet domain proteins contain conserved “aromatic cages” that are predicted to recognize methylated lysine.^13^ However, no Agenet domain-containing histone reader in plants has been characterized yet and the recognition mode of Agenet domain is still a question.

The H3K9 methylation pattern in *Arabidopsis* is different from that in animals. In *Arabidopsis*, H3K9me2 modification is the major mark of gene silencing, while the abundance of H3K9me3 modification is relatively low.^14^ H3K9me2 is also involved in a positive-feedback loop with DNA methylation.^15^ H3K9me2 modification is created by histone methyltransferases SUVH4 (KYP), SUVH5 and SUVH6. SUVH4 contains a SRA domain recognizing the methylated DNA; and the DNA methylation writers, such as CMT3, contain chromodomain and BAH domain recognizing H3K9me2.^15^ H3K9me2 and DNA methylation combinatorically maintain the epigenetic silencing of transposable elements,^16^ thus identifying the H3K9me2 readers that involved in this positive-feedback loop is crucial to fully understand the mechanisms safeguarding genome stability.

The Agenet Domain Containing Protein 1 (ADCP1) is a plant-specific poly-Agenet protein and comprises three tandem Agenet modular domains. In this study, we report on the discovery of ADCP1 as a multivalent histone H3K9 methylation reader in plants, and outline its functional roles in mediating heterochromatin phase separation, H3K9 and CHG/CHH DNA methylation maintenance, as well as transposon silencing. Despite distinct domain compositions, our results suggest that ADCP1 and HP1 are functionally equivalent and have been convergently evolved with similar histone reader activity and multivalent feature for heterochromatin regulation.

## RESULTS

### ADCP1 is an H3K9me2-specific reader weakened by H3S10ph

**Fig. 1:**
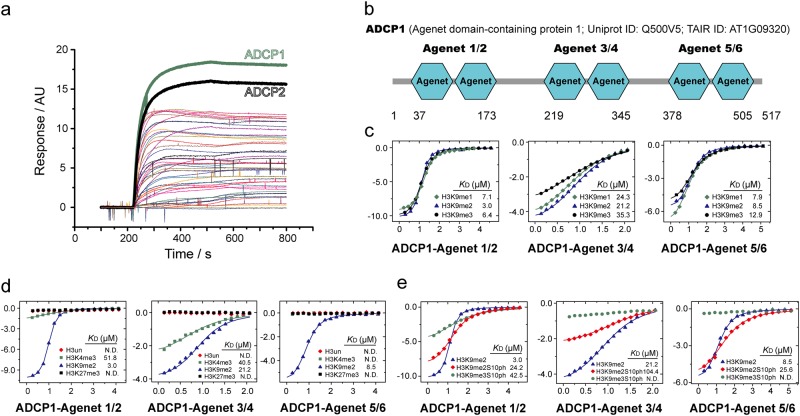
The domain architecture and binding property of ADCP1. **a** Surface plasmon resonance imaging profiling of “Royal family” proteins binding to H3K9me2 peptide. 200 s-500 s was the association phase; 500 s-800 s was the dissociation phase. **b** The domain architecture of ADCP1. **c** The ITC fitting curves of H3(1-15)K9me1/2/3 peptides titrated to three tandem Agenet domains of ADCP1. **d** The ITC fitting curves of histone peptides methylated at different sites titrated to three tandem Agenet domains of ADCP1. **e** The ITC fitting curves of phosphorylated histone peptides titrated to three tandem Agenet domains of ADCP1

To identify the homolog of HP1 in plants, we screened “Royal Family” proteins in *Arabidopsis* through a 3D-carbene based SPRi platform.^17,18^ One Agenet domain-containing protein AT1G09320 (abbreviated as ADCP1) showed a significant signal towards H3K9me2 peptide on the SPRi platform (Fig. 1a). ADCP1 contains three conserved tandem Agenet domains, which are labelled as Agenet 1/2, Agenet 3/4, and Agenet 5/6 (Fig. 1b and Supplementary information, Figs. S1, 2). To determine which tandem Agenet domain(s) in ADCP1 functions as the H3K9me2 reader, we expressed and purified the three tandem Agenet domains separately and performed isothermal titration calorimetry (ITC) experiments. All three tandem Agenet domains could recognize one H3K9me modified histone peptide (Fig. 1c and Supplementary Table S2), rendering full-length ADCP1 a multivalent histone reader like HP1 dimer that recognizes H3K9me3 nucleosomes.^19^ The binding affinities of H3K9me1 and H3K9me3 peptides to ADCP1 were in the same range of the H3K9me2 peptide despite some slight preference for H3K9me2 (Fig. 1c). Thus, ADCP1 is an H3K9me2 reader and does not strictly discriminate on the basis of the methylation states. Notably, in *Arabidopsis*, H3K9me2 but not H3K9me3 functions as the dominant histone H3K9 methylation form that marks heterochromatin.^14^ Hence, we focused on H3K9me2 in the following structural and functional studies.

To explore the histone modification sites specificity of ADCP1, we titrated unmodified H3, H3K4me3 and H3K27me3 peptides to tandem Agenet domains of ADCP1. The results showed that ADCP1 did not bind to unmodified H3 or H3K27me3 and the binding affinities to H3K4me3 were much weaker than those of H3K9me2 (Fig. 1d). Next, we explored whether ADCP1 was sensitive to H3S10ph. Our results showed that adjacent H3S10ph modification weakened the binding affinity of ADCP1 to H3K9me2 peptide (Fig. 1e).

### Structural basis for H3K9me2 recognition by tandem Agenet domains

**Fig. 2:**
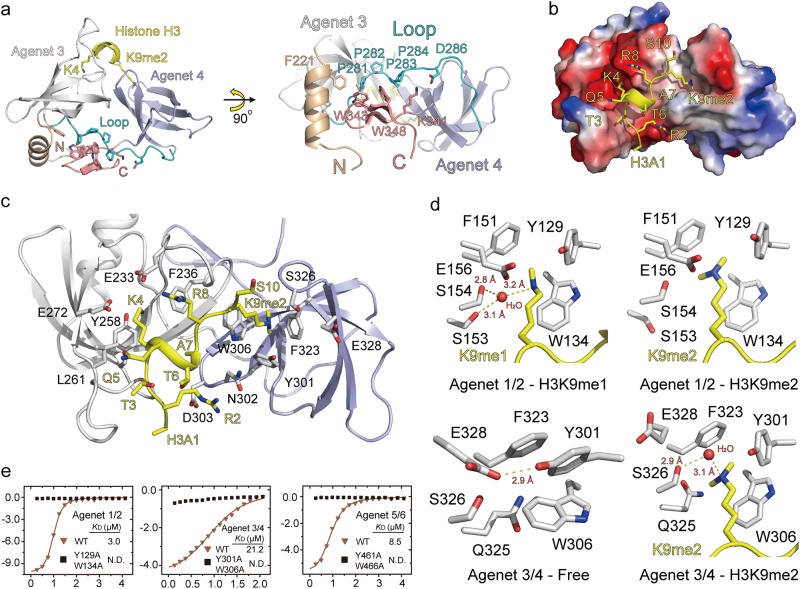
The structural basis of ADCP1 recognizing H3K9me2 modification. **a** The overall structure of ADCP1-Agenet 3/4 domain in complex with H3(1-15)K9me2 peptide. **b** The electrostatic surface view of ADCP1-Agenet 3/4 domain in complex with H3(1-15)K9me2 peptide. **c** The binding details of H3(1-15)K9me2 peptide to ADCP1-Agenet 3/4 domain. **d** The binding pocket of H3K9me1/2 modification in ADCP1-Agenet domains. **e** The ITC fitting curves of H3(1-15)K9me2 peptide to wild type and mutant Agenet domains of ADCP1. N.D., Not Detected

To understand the structural basis for H3K9me2 recognition by ADCP1, we solved the crystal structures of the Agenet 1/2-H3K9me1 complex, Agenet 1/2-H3K9me2 complex, Agenet 3/4-free and Agenet 3/4-H3K9me2 complex at 2.7, 2.7, 2.3 and 1.7 Å, respectively (Supplementary information, Fig. S3a and Table S1). The alignment of tandem Agenet domains revealed that the overall structures of Agenet 1/2 and Agenet 3/4 were conserved and that the peptide binding did not trigger obvious conformational change of ADCP1 (Supplementary information, Fig. S3b). Considering the high-resolution structure of the Agenet 3/4-H3K9me2 complex, we used it as an example to illustrate the binding mode of tandem Agenet domain to H3K9me2 peptide. The complex structure showed that the tandem Agenet domains of ADCP1 adopted a “face-to-face” conformation. The N-terminus and C-terminus of the tandem Agenet domain together with the Pro-rich loop between the two Agenet domains formed a stable hydrophobic core that is accessible for peptide recognition (Fig. 2a). The histone peptide was induced to form a “helix-loop” structure and bound to the cleft between the two Agenet domains (Fig. 2a, b and Supplementary information, Fig. S3c). The H3K9me2 modified histone peptide bound to a negatively charged surface on ADCP1 with H3K9me2 directed towards the second Agenet domain (Fig. 2b). H3K9me2 is inserted into an aromatic cage formed by Y301, W306 and F323 (Fig. 2c). H3R2 was stabilized through interactions with N302 and D303, while H3K4 was recognized by E233 and E272 (Fig. 2c). H3A7, H3R8, and H3S10 are not involved in direct interactions with ADCP1. Histone H3K27 and H3K9 shared the same “ARKS” motif but this motif was not recognized by ADCP1, and hence ADCP1 showed no binding ability towards H3K27me3.

In the free structure of Agenet 3/4, Y301 and E328 formed a hydrogen bond, resulting in closure of the aromatic cage (Fig. 2d). Upon histone binding, the aromatic cage of ADCP1 undergoes conformational adjustments and E328 is flipped out. H3K9me2 forms a water-mediated hydrogen bond with S326 and a cation-π interaction with Y301, W306 and F323. In the Agenet 1/2-H3K9me1 complex, H3K9me1 forms a water-mediated hydrogen bond with S153, S154 and cation-π interactions with Y129, W134, and F151. In the Agenet 1/2-H3K9me2 complex, we did not observe water-mediated hydrogen bond and H3K9me2 was recognized by Y129, W134, and F151 (Fig. 2d). Although reader domain types vary, recognition of the H3K9 methylation by an aromatic cage is a conserved molecular mechanism amongst different readers, such as Agenet, SAWADEE, BAH, Tudor and Chromo domains (Supplementary information, Fig. S4). To confirm that H3K9me2 was recognized by the aromatic cage, we generated double-mutant tandem Agenet domains (Agenet 1/2-Y129A/W134A, Agenet 3/4-Y301A/W306A, Agenet 5/6-Y461A/W466A) and performed ITC experiments. The ITC results demonstrated that these binding pocket mutants of ADCP1 could not bind to H3K9me2 peptide (Fig. 2e).

### ADCP1 is directly associated with heterochromatic regions

**Fig. 3:**
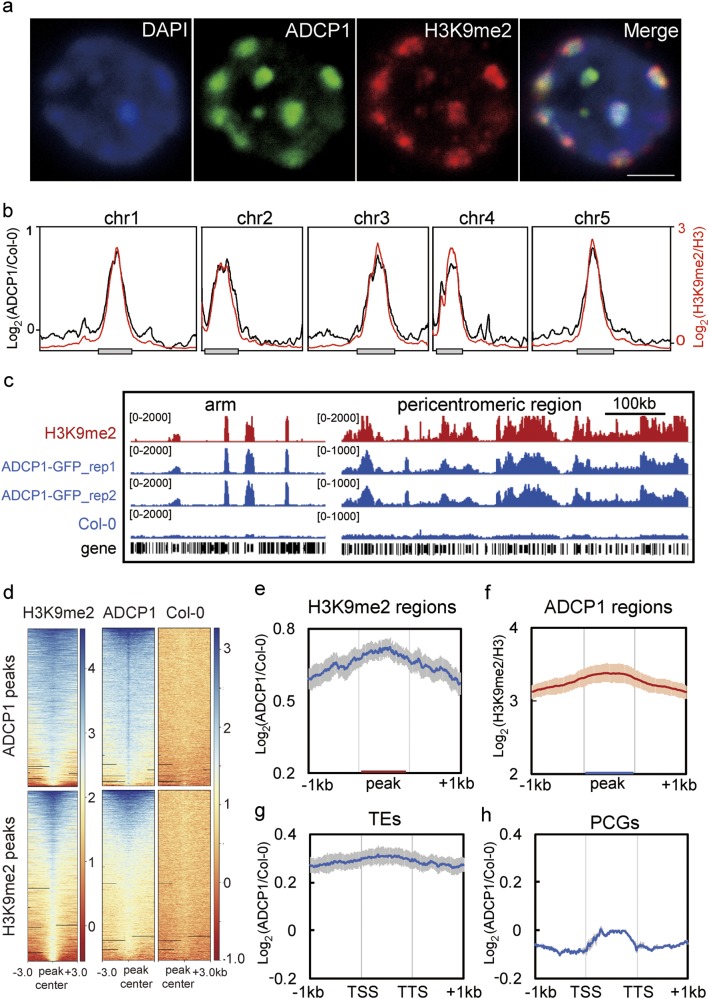
ADCP1 is directly associated with the H3K9me2 heterochromatin. **a** Immunostaining of interphase nuclei with pADCP1:ADCP1: GFP transgenic plants. Colors indicated the DNA counterstained with DAPI (blue), ADCP1-GFP (green), and H3K9me2 (red). Bar = 2μm. **b** Distribution of ADCP1 (black) and H3K9me2 (red) in the 5 *Arabidopsis* chromosomes. The gray boxes indicate the pericentromeric region of each chromosome. The left y axis means log_2_ ratio of ADCP1-GFP ChIP-seq signals to Col-0 and the right y axis means log_2_ ratio of H3K9me2 to H3 ChIP-seq signals in Col-0. The data were plotted with the mean of two biological replicates and smoothed using LOESS method by GraphPad Prism. **c** Snapshots of ADCP1 and H3K9me2 signals in selected chromosome arm (chr3: 9600-9800) and pericentromeric region (chr1: 13900-14500). H3K9me2 and GFP ChIP-seq signals are showed as reads per kilobase per million mapped reads (RPKM). GFP ChIP-seq in Col-0 is shown as the negative control. **d** Heatmaps of H3K9me2 and ADCP1 ChIP-seq enrichment in ADCP1-bound sites (top) and H3K9me2-bound sites (bottom). Each row represents a 6-kb window centred on peak midpoints. ADCP1 and Col-0 are showed as log_2_ (GFP ChIP/input), H3K9me2 is showed as log_2_ (H3K9me2/H3). **e** Metaplots of ADCP1 ChIP-seq signal in H3K9me2-marked regions. **f** Metaplots of H3K9me2 level in ADCP1-enriched peaks. **g-h** Metaplots of ADCP1 ChIP-seq signals in TEs (**g**) and in the protein coding genes (**h**). All metaplots are plotted with mean values and the shadow means the standard deviation of two biological replicates

Next, we sought to determine whether ADCP1 could recognize H3K9me2 in vivo. GUS staining with ADCP1-GUS fused complementation plants showed that ADCP1 was expressed ubiquitously in *Arabidopsis* in the vegetative stage, meristem including root tip and shoot apical meristem, reproductive stage, ovules and early stages of seeds (Supplementary information, Fig. S5a). To determine the detailed localization profile of ADCP1 in the nucleus, we then produced the GFP-fused ADCP1 complementation plants (Supplementary information, Fig. S5b). Consistent with in vitro binding activity, results from immunostaining with anti-GFP antibody showed that ADCP1 strongly co-localized with heterochromatin mark H3K9me2, mainly within the heterochromatin regions (Fig. 3a and Supplementary information, Fig. S5c, d). To further explore the in vivo binding pattern of ADCP1, we performed chromatin-immunoprecipitation followed by sequencing (ChIP-seq) with anti-GFP antibody in the ADCP1-GFP complementation plants. We found that ADCP1 was highly enriched in pericentromeric regions and perfectly co-localized with H3K9me2 (Fig. 3b, c and Supplementary information, Fig. S6a). Besides, ADCP1 showed a significant enrichment on H3K9me2-marked regions, and vice versa (Fig. 3d–f). Permutation test clearly indicated that ADCP1 had a significant correlation with H3K9me2 (Supplementary information, Fig. S6b).^20^

ADCP1 was also enriched in heterochromatic patches on the euchromatic arms, where high levels of H3K9me2 were also observed (Fig. 3c). We also found a strong enrichment of ADCP1 over transposable elements (TEs) (Fig. 3g). The detailed analysis showed that almost all of the ADCP1 targets belonged to TEs, and more than 97% of them were also the targets of H3K9me2 (Supplementary information, Fig. S6c and Table S5). Furthermore, ADCP1 favored large TEs (>4 kb) and retrotransposon, especially LTR/Gypsy (Supplementary information, Fig. S6d, e). In contrast, ADCP1 was not enriched over protein coding genes (Fig. 3h), consistent with previous data that the majority of genes were devoid of H3K9me2.^21^ Collectively, ADCP1 recognizes and associates with H3K9me2-marked heterochromatin regions in vivo, which pointed to the possibility that ADCP1 functions as the Heterochromatin Protein 1 in *Arabidopsis*.

### ADCP1 is required for heterochromatic chromocenter formation

**Fig. 4:**
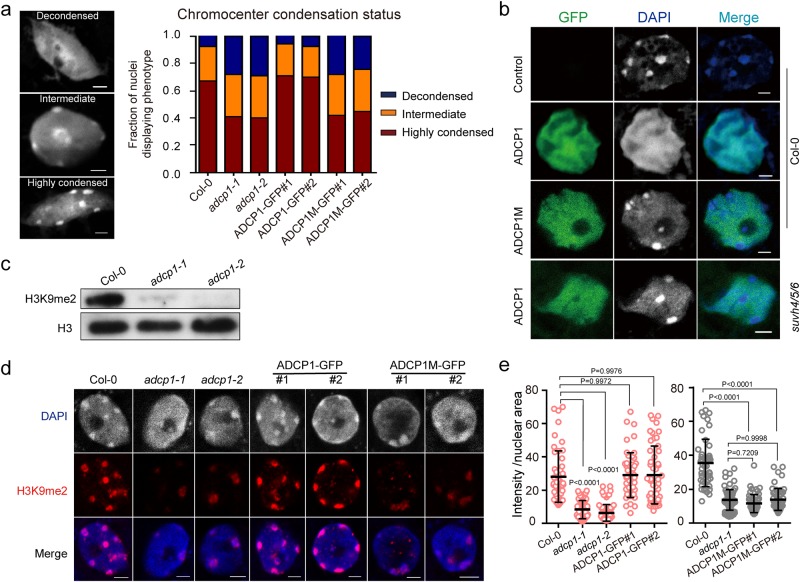
ADCP1 is required for heterochromatin formation. **a** Relative frequencies of decondensed, partially decondensed (intermediate), or wild-type chromocenters in DAPI-stained nuclei of wild-type Col-0, *adcp* mutants and ADCP1/ADCP1M complementary plants. *N* = 150. Representative nuclear condensation status stained with DAPI is showed on the left. Bar = 2 μm. **b** Transient expression of 35 S:ADCP1: GFP or 35 S:ADCP1M: GFP in protoplasts of Col-0 and *suvh456*. After 14 h of protoplasts transformation, GFP signals were observed (showed in green, and DAPI is blue). Bar = 2μm. **c** H3K9me2 western blot in Col-0 and *adcp1* mutants. **d** H3K9me2 immunostaining in Col-0, *adcp* mutants and ADCP1/ADCP1M complementary plants. Bar = 2 μm. **e** Quantification of H3K9me2 immunostaining in panel **d**, the intensity is normalized to the nuclear area. *N* = 50. The black line represents mean ± SD. The red circles and gray circles are two independent experiments. *P* values from one-way ANOVA analysis are reported. Three biological replicates were performed for all figures

To assess the function of ADCP1, we isolated a homozygous T-DNA insertion mutant *adcp1-1*, and generated a 1 kb deletion mutant *adcp1-2* with CRISPR-Cas9 system (Supplementary information, Fig. S7a, b). We observed that the proportion of decondensed nuclei was largely increased in *adcp1* mutants compared to that in Col-0, and this observation was rescued when *adcp1-1* was complemented with ADCP1-GFP (Fig. 4a and Supplementary information, Fig. S7c). This result indicates that ADCP1 is required for the organization of heterochromatic chromocenters. In order to test whether the binding ability of ADCP1 to H3K9me2 was required for heterochromatic chromocenters formation, we mutagenized all three H3K9me2 binding pockets of ADCP1 (ADCP1M, Y129A/W134A/Y301A/W306A/Y461A/W466A) in the ADCP1 complementation construct, which disrupted the binding ability of ADCP1 to H3K9me2 (Fig. 2e). The complementation of ADCP1M-GFP in *adcp1-1* could not rescue the increased ratio of decondensed nuclei (Fig. 4a and Supplementary information, Figs. S5b, 7c). To further validate this function, we performed transient protoplast transformation with ADCP1 overexpression construct (35 S:ADCP1: GFP). After 14 h expression, we fixed the protoplasts and counterstained the nuclei with DAPI. The ADCP1 expression level was too high so that we cannot observe discrete nuclear puncta for ADCP1 localization (Supplementary information, Fig. S7d). Interestingly, we observed that the nuclear chromocenters became diffused when ADCP1 was transiently overexpressed, and the stained nuclear DNA distribution was consistent with ADCP1 signals, suggesting a role of ADCP1 (in proper dosage) in chromocenter formation (Fig. 4b and Supplementary information, Fig. S7d). By contrast, the nuclear condensation states of protoplasts transformed with ADCP1M overexpression construct showed the same pattern as the untransformed controls (Fig. 4b), indicating that the H3K9me2 readout is regulated by ADCP1 in this process. Next, we transformed ADCP1 to the protoplasts prepared from *suvh456*, the triple mutant that lost the H3K9me2 modification globally (Supplementary information, Fig. S7e). The nuclear signals of *suvh456* with ADCP1-GFP display a slightly blurry morphology due to the lack of H3K9me2 (Fig. 4b). Notably, the signals of both ADCP1M in Col-0 and ADCP1 in *suvh456* are excluded from the chromocenters (Fig. 4b), consistent with a critical role of H3K9me2 recognition in determining the nuclear distribution of ADCP1. Finally, we checked the H3K9me2 pattern in Col-0 that overexpressed ADCP1 and ADCP1M by immunostaining. It appeared that the disordered nuclei caused by ADCP1 overexpression was not accompanied by loss of the H3K9me2 foci, yet speckles of H3K9me2 are less focused in the case of ADCP1 but not ADCP1M (Supplementary information, Fig. S7f). Collectively, these results showed that ADCP1 is required for maintaining the heterochromatin patterning in the nuclei, and this function likely depends on its binding ability to H3K9me2.

### ADCP1 regulates H3K9/CHG/CHH methylation levels and TE silencing

**Fig. 5:**
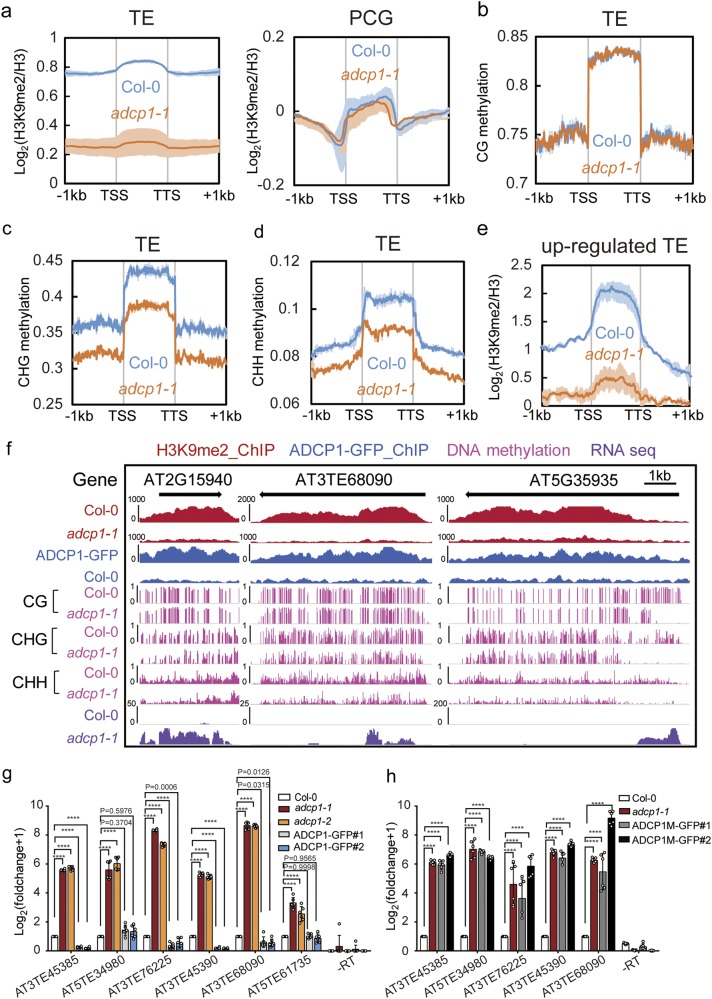
ADCP1 regulates H3K9me2 and CHG/CHH methylation levels and TE silencing. **a** Metaplots of H3K9me2 level in TEs (left) and protein coding genes (PCG, right) in Col-0 and *adcp1-1*. The shadow means the standard deviation of two biological replicates. **b**–**d** Metaplots of CG methylation (**b**), CHG (**c**) and CHH (**d**) in TEs. **e** Metaplot of H3K9me2 level in up-regulated TEs. All metaplots are plotted with mean values. The shadow means the standard deviation of two biological replicates. **f** Snapshots of all sequencing data at three selected transposon elements. ChIP-seq and RNA-seq signals are shown as RPKM, DNA methylation is shown by the ratio of methylated cytosine to all cytosine. **g**, **h** Validation of selected up-regulated TEs in Col-0, *adcp* mutants and ADCP1/ADCP1M complementary plants by RT-qPCR. PCR using primers of AT3TE6809 with templates of minus RT (-RT) was used as a negative control. The relative expression to *UBC* and *GAPDH* gene is normalized to that in Col-0. Data were shown as log_2_ (fold change + 1). Error bars indicate standard deviations of 6 replicates, including 2 biological replicates with 3 technical replicates. The circles represent the original data. One-way ANOVA was used for the statistical analysis, *P* < 0.0001(****)

Since the morphology of heterochromatin was affected in *adcp*1 mutants, we were then prompted to check the level of H3K9me2 and DNA methylation. Firstly, we extracted total histones and performed immunoblot of H3K9me2. A significant decrease of the H3K9me2 level was observed in *adcp1* mutants compared to that in Col-0 (Fig. 4c). Quantification of H3K9me2 immunostaining was also performed. We observed a clear reduction of the H3K9me2 level in *adcp1* mutants, and this could be complemented to the level of wild type upon ADCP1 re-expression, while this couldn’t be restored upon ADCP1M re-expression (Fig. 4d, e). Consistent with these results, the native ChIP-seq data also displayed genome-wide decreased H3K9me2 level in *adcp1* mutants, especially in the pericentromeric regions that were enriched with TEs (Fig. 5a and Supplementary information, Fig. S8a, b); this result was also validated by ChIP-qPCR within the selected loci (Supplementary information, Fig. S8c). To see whether genomic DNA methylation was influenced in *adcp1* mutants, we performed the whole-genome bisulfite sequencing in the wild type and mutant plants. Over TEs, the total levels of DNA methylation at CHG/CHH sites were significantly reduced but rarely eliminated in *adcp1* mutants, while DNA methylation at CG sites remained unchanged (Fig. 5b, c, d and Supplementary information, Fig. S8d-g). Our result suggest that ADCP1 is required for the maintenance of H3K9me2 and CHG/CHH methylation levels in vivo.

Heterochromatin formation is also important for transposon silencing and genome stability maintenance. In order to explore the function of ADCP1 in gene and TE silencing, an RNA-seq experiment was performed in the wild type and mutant plants. In the *adcp1-1* mutant, 70% of the upregulated TEs were the targets of H3K9me2 and showed a largely decrease of H3K9me2 (Fig. 5e, f and Supplementary information, Fig. S8h). Besides, most of the depressed TEs belong to retrotransposons (Fig. 5f and Supplementary information, Table S6), which may have transposition activity and be dangerous to genome stability. RT-qPCR results confirmed their up-regulated expression in *adcp1* mutants, and the reactivation of TE expression in *adcp1* mutants could be repressed in the ADCP1 complementation plants (Fig. 5g), but not in plants complemented with the ADCP1M mutant (Fig. 5h). These results suggest that ADCP1 plays an important role in transcriptional silencing of TEs through modulating the level of DNA and H3K9 methylation.

### ADCP1 mediates heterochromatin phase separation

**Fig. 6:**
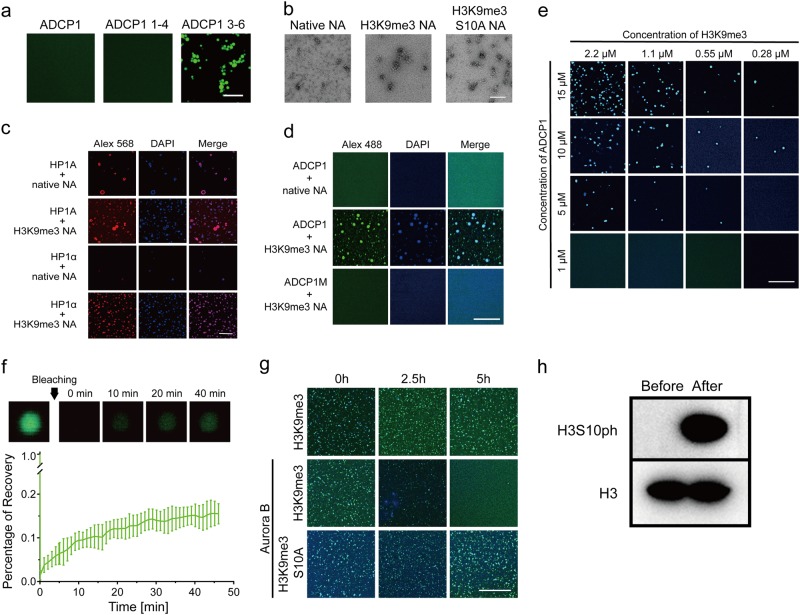
ADCP1 drives multivalent H3K9me3 nucleosome array phase separation. **a** The phase separation of ADCP1 full length, Agenet domain 1-4 and Agenet domain 3-6 protein only, bar = 20 μm. **b** Electron microscope image of native, H3K9me3 and H3K9me3S10A NA, bar = 100 nM. **c** Droplets formation of HP1A, HP1αwith H3K9me3 NA. HP1A, HP1α were labelled with Alex 568, while the NA was strained by DAPI. Bar = 20 μm. **d** Phase separation of ADCP1 with H3K9me3 NA. ADCP1 was labelled by Alex 488, while the NA was strained by DAPI. Bar = 20 μm. **e** The concentration dependence of phase separation. Bar = 20 μm. The merged images with Alex 488 fluorescence and DAPI staining were shown. **f** The FRAP of the droplets. Five droplets were bleached with 488 laser and the percentage of recovery was recorded. One of the five droplets was imaged to show the dynamic. **g** The phase separation of Aurora B treated H3K9me3 NA and H3K9me3S10A NA with ADCP1. Bar = 20 μm. **h** Western blots of H3K9me3 NA before and after aurora B treatment

Recent studies have shown that human HP1α (upon phosphorylation) and fly HP1a have intrinsic, weak phase transition potentials,^9,10^ and HP1α’s C-terminus negatively regulates the phase transition ability of HP1α.^9^ Interestingly, we observed that a truncated form containing Agenet 3-6 but not full length ADCP1 has intrinsic phase transition potential (Fig. 6a), suggesting the fragment containing Agenet 1-2 also negatively regulates the phase transition ability of ADCP1. The work on animal HP1 suggests a role of phase separation in heterochromatin formation.^9,10^ To biochemically confirm this, we prepared methyl-lysine analog (MLA)^22^ histone H3K9me3 and reconstituted a “designer” nucleosome array (NA) with MLA H3K9me3 marks (Fig. 6b). Consistently, we showed that H3K9me3 NA promotes phase transition of HP1α and HP1a in comparison with native NA (Fig. 6b, c). Given the observation that ADCP1 and H3K9me2 co-localize to chromocenters in plant cells (Fig. 3a), similar to HP1 and H3K9me3 in animal cells,^23^ we wondered whether ADCP1 is also involved in phase separation of H3K9me3 NA. Indeed, ADCP1 forms DNA-rich puncta with H3K9me3 NA but not with native NA in vitro (upper-middle panels, Fig. 6d). Puncta formation is H3K9me recognition-dependent as a reader pocket mutant ADCP1 failed to form puncta with H3K9me3 NA (lower panels, Fig. 6d). Several lines of evidence suggest that the puncta formation by ADCP1/H3K9me3 NA is due to phase transition: (1) the morphology of puncta is nearly a perfect round/sphere (Fig. 6d); (2) puncta formation shows sharp concentration dependence on both components (Fig. 6e); (3) ADCP1 was dynamically exchanged between puncta and ambient solution as its fluorescence recovers after being bleached (Fig. 6f).

Similar to a phosphorylation-dependent binary switch mechanism that has been observed for HP1,^6,7^ ITC titration showed that adjacent H3S10ph can also switch off binding between ADCP1 and H3K9me2/3 (Fig. 1d). Next we tested the responsiveness of phase-separated ADCP1-H3K9me3 NA to H3S10 phosphorylation. Both the H3K9me3 NA and the H3(S10A)K9me3 NA that harbors H3S10 alanine mutation form phase transition with ADCP1. However, phase transition of the former but not the latter can be dissolved by Aurora B treatment that causes S10 phosphorylation (Fig. 6g, h).

Collectively, the above results show that ADCP1 functions as an HP1 equivalent protein in plant to regulate heterochromatin formation through a conserved phase separation mechanism.

## DISCUSSION

HP1 in animals is crucial for heterochromatin formation and functions. However, LHP1, the homolog of HP1 in plants, functionally recognizes H3K27me3 and better resembles Polycomb proteins.^11,12^ In contrast, the ubiquitously expressed ADCP1 binds to H3K9me2 both in vitro and in vivo and is sensitive to H3S10ph. Structurally, ADCP1 induces a “helix-loop” structure of the H3(1-10) peptide and recognizes the “R2-K4-K9me2” motif instead of the “ARKS” motif shared by H3K9 and H3K27, rendering it a specific H3K9me2 reader. For animal HP1-H3K9me2/3 interactions, the histone tail inserts as an induced β-strand to complete a β-sandwich architecture of the chromodomain, and the H3 “Q5-T6-A7-R-8-K9me2/3-S10” motif mediates most interactions.^24,25^ Despite distinct motif recognition mechanisms, both HP1 chromodomain and ADCP1 tandem Agenet adopt a similar aromatic cage for H3K9 methylation readout.

The normal expression of ADCP1 is crucial for the heterochromatin maintenance, suggesting its role in organizing heterochromatin. The knock-out of *adcp1* leads to global decrease of methylation levels of histone H3K9 and DNA CHG/CHH sites, indicating that ADCP1 is involved in the positive feedback loop of establishing H3K9me2 and DNA methylations.^15^ Knock-out of *adcp1* also leads to the activation of a repertoire of transposable elements, stressing its role in TE silencing and genome stability maintenance. Importantly, we further showed that phenotypic defects caused by *adcp1* knock-out could be rescued by re-introducing the wild type *adcp1* but not its reader deficient mutant. Collectively, these results suggest that ADCP1 is a key H3K9me2 reader that regulates DNA methylation and heterochromatin formation in plants.

Agenet domain is one of the “Royal family” members and abundant in plants (Supplementary Fig. S1), but its histone reader activity has not been well characterized. *Arabidopsis* ADCP1 contains three tandem Agenet domains and all these three tandem Agenet domains recognize H3K9me2. One protein contains more than one histone reader modules can empower its multivalent engagement of histone modification patterns.^26^ For example, the presence of “PHD-Bromo” cassette in BPTF leads to the combinatorial readout of “H3K4me3-H4Kac” modification patterns at the nucleosomal level.^27^ ADCP1 is a unique histone reader that recognizes one type of histone modification trivalently. In addition, we showed that Agenet 3-6 of ADCP1 has intrinsic phase separation ability that is inhibited and thus regulated by the presence of the Agenet 1-2. Such a feature is reminiscent of human HP1α, in which a very C-terminal region of HP1α negatively regulates the intrinsic phase transition potential of an N-terminally phosphorylated HP1α.^9^ The multivalent and intrinsic phase transition features of ADCP1 likely render it a good scaffold protein in organizing biomolecular phase separation.^28^ Conceivably, through the formation of dynamic nuclear condensates,^29^ the phase-separated ADCP1 may promote the recruitment of additional client chromatin regulators such as SUVH4 or CMT3 to maintain H3K9 and DNA methylation levels. HP1 was reported to promote heterochromatin formation through H3K9me3 recognition, which could be reversed by adjacent H3S10ph.^6,7^ Using reconstituted designer nucleosome array as a probe, we showed that plant ADCP1 could drive phase separation in vitro in an H3K9me3-dependent manner. Remarkably, such phase transition could be switched off by additional H3S10 phosphorylation catalyzed by Aurora B, suggesting a “phospho-switch” mechanism for down-regulation of the ACDP1-mediated phase separation.

In sum, ADCP1 shows highly common features with HP1 in its histone binding activity, epigenetic silencing function and phase transition capability despite their distinct domain compositions. Based on these properties of ADCP1, we hold the view that ADCP1 rather than LHP1 constitutes the HP1 equivalent in plants to regulate heterochromatin formation. The mechanistically convergent evolution of two distally related proteins for a consensus function calls attention to the complexity and conservation of epigenetic regulation among different kingdoms of life. Considering the wide distribution of ADCP1 orthologues in plants, we envision that our studies shall pave the way for many new initiatives centering on the role of the Agenet domain family members in epigenetic regulation and beyond.

## MATERIALS AND METHODS

### Plasmids and plant strains

The cDNAs encoding the Agenet 1/2 domain (amino acids 31-177), Agenet 3/4 domain (amino acids 201-355), Agenet 5/6 domain (amino acids 376-517), Agenet 1-4 domain (amino acids 1-355) and Agenet 3-6 (amino acids 201-517) of ADCP1 were cloned into the pGEX6p vectors (GE Healthcare). Full length ADCP1 was cloned into the pRSFDuet vectors with a C-terminal His-tag. Point mutations were generated using the QuikChange site-directed mutagenesis kit (Stratagene). Histone peptides bearing different modifications were synthesized at SciLight Biotechnology.

Seeds of wild-type *Arabidopsis* ecotype Col-0 (Columbia-0) and T-DNA insertion mutant *adcp1-1* (SALK_130936) were obtained from the Nottingham *Arabidopsis* Stock Centre, UK. Genotyping primers are listed in Supplementary information, Table S3. The plant CRISPR/Cas9 system^30^ was used to generate the genomic deletion of ADCP1 gene (*adcp1-2*). The sequences of two sgRNAs are listed in Supplementary information, Table S3, and the locations of these sgRNAs are indicated in Supplementary information, Fig. S7a. Sequencing analysis of 2 individual mutations of *adcp1-2* are shown in Supplementary information, Fig. S7b. *suvh456* was previously described.^31,32^ The overexpression plasmids were constructed by cloning the CDS of ADCP1 into PUC19 vectors containing 35S promoter and eGFP tag. For complementation experiments, *ADCP1* genomic DNA from 2 kb upstream of ATG to 1 kb downstream of TAG were cloned into pBar1001 (modified based on pCAMBIA1300 construct with replacement of plant resistance marker Hygromycin to Basta) and the GUS/GFP tags were fused to the N-terminal of ADCP1. These vectors were constructed by Fast-Cloning method^33^ and the primers are listed in Supplementary information, Table S3.

### Protein expression and purification

The tandem Agenet domains of ADCP1 (Agenet 1/2, Agenet 3/4, Agenet 5/6, Agenet 1-4 and Agenet 3-6) were cloned into pGEX6p vector and expressed with an N-terminal GST-tag in *Escherichia coli* strain BL21 (DE3) in the presence of 0.4 mM IPTG. Overnight-induced cells were collected by centrifugation and re-suspended in lysis buffer: 100 mM NaCl, 20 mM Tris-HCl, pH 7.5. Then cells were lysed with an Emulsiflex C3 (Avestin) high-pressure homogenizer. After centrifugation at 16,770 × g, the supernatant was applied to a GST column, and proteins were directly digested on the column overnight by the PreScission proteases. The resultant protein was further purified by anion-exchange chromatography using a HiTrap Q column (GE Healthcare). The peaks eluted were applied to a Superdex 75 10/300GL (GE Healthcare) gel filtration column. The Se-methionine-substituted Agenet 3/4 protein was expressed in M9 medium supplemented with amino acids Lys, Thr, Phe, Leu, Ile, Val and Se-Met, and purified using the same protocol as the wild-type protein.

Full-length MBP-ADCP1-His was loaded on HisTrap column (GE Healthcare). The protein was eluted with the buffer 100 mM NaCl, 20 mM Tris-HCl, pH 7.5, 500 mM imidazole and the N-terminal MBP tag was removed with TEV protease. Full-length ADCP1-His was loaded on HisTrap column and the eluted peak was applied to Q column. The eluted protein was applied to a Superdex 200 10/300GL (GE Healthcare) gel filtration column.

All proteins were stored in 100 mM NaCl, 20 mM Tris-HCl, pH 7.5, at ~ 10 mg/ml in a −80 °C freezer. Mutants were expressed and purified using essentially the same procedure as for the wild-type proteins.

### Protein crystallography

Crystallization was performed by the sitting-drop vapor diffusion method under 18 °C by mixing equal volumes (1–2 μL) of protein and reservoir solution. Free Agenet 3/4 (8-10 mg/mL) crystals were grown in the solution containing 2.1 M DL-Malic acid pH 7.0. Crystals were directly flash frozen in liquid nitrogen for data collection at 100 K. For complex crystallization, Agenet 1/2 (8–10 mg/ml) and Agenet 3/4 (8–10 mg/ml) were first incubated with histone peptides (SciLight Biotechnology) at molar ratio of 1:5 for about 1 h. Crystals of Agenet1/2-H3K9me1 and Agenet1/2-H3K9me2 could be obtained under 28% (w/v) PEG 4000, 0.1 M sodium acetate, pH 5.0, 0.2 M ammonium acetate. Crystals were briefly soaked in a cryo-protectant composed of reservoir solution supplemented with 8% glycerol, and were flash frozen in liquid nitrogen for data collection at 100 K. Crystals of Agenet 3/4-H3K9me2 could be obtained under 2.0 M ammonium formate, 0.1 M HepesNa, pH 7.5. Crystals were directly flash frozen in liquid nitrogen for data collection at 100 K. All data sets were collected at beamline BL17U at the Shanghai Synchrotron Radiation Facility at 0.9793 Å. All data were indexed, integrated and merged using the HKL2000 software package.^34^

The phase of free Agenet was solved by the single-wavelength anomalous dispersion method using PHENIX software. The structures of Agenet 1/2-H3K9me1, Agenet 1/2-H3K9me2 and Agenet 3/4-H3K9me2 complexes were solved by molecular replacement using MOLREP^35^ with the free Agenet 3/4 structure as a search model. All structures were refined using PHENIX,^36^ with iterative manual model building using COOT.^37^ Detailed structural refinement statistics are shown in Supplementary information, Table S1. All structural figures were created using PYMOL (http://www.pymol.org/).

### Surface plasmon resonance imaging

To measure the interaction between immobilized peptides and flowing proteins, an SPR imaging instrument (Kx5, Plexera, USA) was used to monitor the whole procedure in real-time. Briefly, a 3D-carbene chip with well-prepared biomolecular microarray was assembled with a plastic flow cell for sample loading. The protein samples were prepared at determined concentrations in TBS running buffer (100 mM NaCl, 20 mM Tris-HCl, pH 7.5) while a 10 mM glycine-HCl buffer (pH 2.0) was used for regeneration. A typical binding curve was obtained by flowing protein sample at 2 μL/s for 300 s’ association and then flowing running buffer for 300 s’ dissociation, followed by 200 s regeneration buffer at 3 μL/s. All the binding signals were converted to standard refractive units (RU) by calibrating every spots with 1% glycerol (w/v) in running buffer with known refractive index change (1200 RU). Binding data were collected and analyzed by a commercial SPRi analysis software (Plexera SPR Data Analysis Model, Plexera, USA).

### Isothermal titration calorimetry

For ITC measurement, synthetic histone peptides (SciLight Biotechnology) and the wild-type and mutant ADCP1 tandem Agenet domains were extensively dialyzed against ITC buffer: 100 mM NaCl, 20 mM Tris-HCl, pH 7.5. The titration was performed using a MicroCal PEAQ-ITC system (Malvern Instruments) at 25 °C. Each ITC titration consisted of 17 successive injections with 0.4 μL for the first and 2.4 μL for the rest. Usually, H3 peptides at 1 mM were titrated into ADCP1 tandem Agenet domains at 0.05–0.1 mM. The resultant ITC curves were processed using Origin 7.0 software (OriginLab) according to the ‘One Set of Sites’ fitting model.

### Immunostaining and nuclear DNA staining

Immunostaining was modified based on previously described methods.^38^ Briefly, 1 g leaves were fixed by 4% paraformaldehyde for 20 min with vacuum and chopped in a petri dish containing 3 mL NEB1 (10 mM Tris-HCl pH 9.5, 10 mM KCl, 500 mM sucrose, 0.1% mercaptoethanol, 0.1% Triton-X 100) with scissors to get homogenate on ice. Then the homogenate was filtered with 40 μm cell strainer and centrifuged 3 min at 2500 rpm at 4℃. The pellet was re-suspended gently in 300 μL NEB2 (10 mM Tris-HCl, pH9.5, 10 mM KCl, 125 mM sucrose, 0.1% mercaptoethanol, 0.1% Triton-X 100) and loaded on the top of 600 μL NEB3 (10 mM Tris-HCl, pH 9.5, 10 mM KCl, 850 mM sucrose, 0.1% mercaptoethanol, 0.1% Triton-X 100). Centrifugation was performed at top speed at 4°C for 30 min to precipitate the nuclei. The nuclei were resuspended with 40 μL NEB1 and spread onto a slide.

For nuclear DNA staining, the slide was mounted with DAPI Fluoromount-G^®^ (SouthernBiotech, 0100-20) and observed under confocal (Zeiss LSM780). 150 nuclei were counted from each genotype.

For immunostaining, the air-dried slide was mounted with 4% paraformaldehyde for 30 min at room temperature. After washing with PBST, the slide was blocked with 5% BSA for 1 h at 37 °C. Then the slide was incubated with primary antibody (1:100; anti-GFP, Abcam, ab290; anti-H3K9me2, Abcam, ab1220) overnight at room temperature. The slide was washed with PBST and incubated with second antibody (1:100; Goat Anti-Mouse IgG H&L (Alexa Fluor^®^ 488), Abcam, ab150113; Goat Anti-Rabbit IgG H&L (Alexa Fluor^®^ 555), ab150078) for 1 h at 37 °C. The slide was washed with PBST and mounted with DAPI Fluoromount-G^®^ for confocal observation (Zeiss LSM780).

### Western blot of histones

Histone was extracted from 14-day-old leaves with the following protocols. Nuclei were extracted using Honda buffer (2.5% Ficoll 400, 5% Dextran T40, 0.4 M sucrose, 25 mM Tris–HCl, pH 7.4, 10 mM MgCl_2_, 10 mM β-mercaptoethanol, 0.5 mM PMSF, protease inhibitor cocktail) and lysed with histone extraction buffer (10 mM Tris-HCl, pH 7.5, 2 mM EDTA, 0.25 M HCl, 5 mM 2-mercaptoethanol β-ME, 0.2 mM PMSF). The nuclei lysis was centrifuged and the supernatant was precipitated with 25% TCA. The precipitated pellets were washed by acetone and dissolved by sample lysate (50 mM Tris-HCl pH7.4, 150 mM NaCl, 1% NP-40). The extracted histones in 2 × SDS sample buffer were boiled and loaded into SDS-PAGE. Samples were separated by SDS-PAGE, blotted onto PVDF membranes, blocked with 5% milk and subsequently incubated with anti-H3 antibodies (1:5000 dilution; Abcam, ab1791), anti-H3K9me2 (1:2000 dilution; Abcam, ab1220). HRP-linked goat anti-mouse antibodies and HRP-linked goat anti-rabbit antibodies (EASTBIO, BE0101, BE0102) were used as secondary antibodies. ECL prime western blotting detection reagent (GE) was used to induce chemiluminescence.

### Protoplast transformation

Protoplast transformation was performed as follows.^39^ Briefly, protoplasts were extracted from 4-weeks-old leaves and transformed with plasmid mediated by 20% PEG-Ca. After 14 h of transient expression, protoplasts were fixed with 4% paraformaldehyde and stained with DAPI.

### RT-qPCR and RNA-seq

For RNA-seq, total RNA was extracted from 14-day-old leaves using Trizol (Ambion). Libraries for two biological replicates of each genotype were constructed and sequenced according to the manufacturer’s protocol (NEBNext^®^ UltraTM Directional RNA Library Prep Kit for Illumina^®^). The rRNA was depleted from total RNA using Ribo-ZeroTM rRNA removal Kit (Illumina). Illumina libraries were generated and sequenced using a 2 × 150 paired-end (PE) configuration on the HiSeq instrument (Illumina). For RT-qPCR, extracted RNAs were treated with DNase I for 15 min, and the cDNA was synthesized by PrimeScript™ RT reagent Kit with gDNA Eraser (Takara, RR047A). Resulting cDNA was used for qPCR amplification using LightCycler 480 SYBR Green I Master (Roche, 4887352001) and primers are listed in Supplementary Table S3. The *UBC* and *GAPDH* genes were included as internal controls for normalization.

### ChIP-qPCR and ChIP-seq

For H3K9me2 ChIP, nuclei were extracted from 2.5 g two-week-old leaves and washed with Honda buffer. The extracted nuclei were re-suspended in MNase digestion buffer (50 mM Tris-HCl, pH 7.6, 5 mM CaCl_2_, 0.1 mM PMSF, 1 × cocktail protease inhibitor) including 5 μL RNase A (Takara, 2158), and incubated for 30 min at 37 °C. Next, Micrococcal Nuclease (NEB, M0247S) digestion was performed and stopped by adding EDTA to a final concentration of 10 mM. Next the nucleosomes were released by adding 0.005% SDS and rotation at 4 °C for 3 h. After centrifugation for 10 min at max speed, the supernatant was diluted and ready for IP. 50 μL Dynabeads Protein G magnetic beads (Invitrogen, 10004D) coupled with 5 μL antibody (anti-H3K9me2, ab1220; anti-H3, ab1791) were added to 1 mL sample and the mixture was rotated at 4 °C overnight. After washed by 50 mM NaCl Wash Buffer,100 mM NaCl Wash Buffer,150 mM NaCl Wash Buffer (50 mM Tris-HCl, pH 7.6, 10 mM EDTA, 50/100/150 mM NaCl, 0.1 mM PMSF, 1× cocktail protease inhibitor), TE Buffer, 10 min for each wash; the DNA was purified by phenol-chloroform for sequencing and qPCR.

For ADCP1 ChIP-seq, the protocol was modified based on previous published method.^40^ Nuclei were extracted from 3 g 14-day-old leaves of ADCP1-GFP complementation plants (Col-0 as a control) and cross-linked in 1% formaldehyde. After stopped by 0.125 M glycine and washed by NRBT, the nuclei were suspended by nuclei lysis buffer (50 mM Tris-HCl, pH 8.0, 10 mM EDTA, 1% SDS, 1 × protease inhibitor). Then the nuclei were sonicated 3 times for 10 min (30 s on/off intervals) using the Bioruptor^®^ Pico. The chromatin was centrifuged at max speed for 10 min and the supernatant was collected. The supernatant was diluted 10 times with ChIP dilution buffer (1.1% Triton X-100, 1.2 mM EDTA, 16.7 mM Tris-HCl, pH 8.0, 167 mM NaCl), and then anti-GFP antibody (Abcam, ab290) with a ratio of 1:500 was added for incubation. After rotating at 4 °C overnight, 50 μL Dynabeads Protein G magnetic beads (Invitrogen, 10004D) washed by ChIP dilution buffer was added to the sample. After rotating for 3 h, the beads were washed sequentially with Low Salt Buffer, High Salt Buffer, LiCl Buffer, TE Buffer (each buffer for a quick wash, and a 5 min wash). 100 μL 10% Chelex resin (Biorad, 1422842) was added to elute immune complexes. The mixture was incubated at 95 °C for 10 min at 1300 rpm, cooled to RT and incubated with 2 μL Protease K (10 mg/mL, Amresco 0706) for 1 h at 45 °C, and boiled for another 10 min at 95 °C and 1300 rpm. Then the elution was purified with phenol-chloroform for sequencing.

The ChIP-seq DNA was sonicated to 200 bp using The Covaris^®^ S220 System and the library was constructed using ACCEL-NGS^®^1 S PLUS DNA LIBRARY KIT (Swift Biosciences, Cat. No. 18096) according to the manufacturer’s instruction.

### Whole genome bisulfite sequencing (BS-seq)

DNA was extracted from 14-day-old leaves with CTAB. After complete bisulfite conversion of 2 μg DNA with EZ DNA Methylation-Gold Kit (Zymo Research,D5005), the DNA was sonicated to 250 bp (Covaris) and subjected to library generation following Illumina’s manufacturer Instructions (Swift Biosciences, ACCEL-NGS^®^ METHYL-SEQ DNA LIBRARY KIT, 30024).

### Data analysis

All libraries were sequenced on the Illumina HiSeq-PE150 platform with paired-end 150 bp read length. Base calling and FastQC were performed with standard Illumina software and then the clean reads were mapped to the *Arabidopsis* genome TAIR10 (http://www.arabidopsis.org/).

For RNAseq, reads were mapped with hisat2,^41^ cufflinks and cuffdiff^42^ were used to find different expressed genes. Different expressed elements in wild type and mutants were defined by applying log_2_ (mutant /wild-type) >1 and *p* < 0.05 cutoffs. The DEGs are listed in Supplementary information, Table S7.

ChIP-seq reads were mapped with Bowtie2, allowing up to one mismatch. Only uniquely mapped reads were retained. ChIP binding peaks were called by MACS2.^43^ DNA reads from ADCP1-GFP ChIP with transgenic plants were normalized with the ones from control ChIP with Col-0 control. ChIP reads for histone modifications were normalized with the ones of H3 ChIP, and only peaks with FDR < 0.05 were identified as enriched regions. Searches for enriched DNA motifs were performed using MEME-ChIP^44^ and Homer^45^ with default parameters (Supplementary information, Fig. S6f and Table S4).

BS-seq reads were mapped by Bismark^46^ with default parameters. After removal of the PCR duplicates, the methylation information was obtained using Bismark with a cutoff of at least 4 coverage depth.

The alignments were converted to bigwig files using deepTools^47^ and imported into the Integrative Genomics Viewer (IGV)^48^ for visualization.

### Statistical analysis

Data were processed using Prism v.6 (GraphPad Software). Comparisons between multiple groups were analyzed by one-way ANOVA test. For qPCR, asterisks indicate significant differences compared to Col-0. ****P < 0.0001. The uncertainty in the mean is reported as the standard deviation of the mean (SD).

### Preparation of histone proteins and DNA template

Recombinant human wild-type histone H2A, H2B, H4 proteins, and mutant H3 (H3C110A, H3K9C/C110A, H3K9C/S10A/C110A) proteins were over-expressed in *E. coli* and purified essentially as described previously.^49^

Plasmid of DNA template harboring 12 × 177 bp tandem repeats of Widom 601 sequence was a gift from Dr. Guohong Li in Institute of Biophysics, Chinese Academy of Sciences. Preparation of 12 × 177 bp 601 DNA template followed the method described previously.^50^ Sequence for the 177 bp DNA repeats was listed as below with 601 DNA sequence underlined:

GAGCATCCGGATCCCCTGGAGAATCCCGGTGCCGAGGCCGCTCAATTGGTCGTAGACAGCTCTAGCACCGCTTAAACGCACGTACGCGCTGTCCCCCGCGTTTTAACCGCCAAGGGGATTACTCCCTAGTCTCCAGGCACGTGTCACATATATACATCCTGTTCCAGTGCCGGACCC

### Methyl-lysine analog (MLA) histone H3K9me3 preparation

Due to lack of sufficient H3K9me3 histone samples, we adopted the methyl-lysine analog (MLA) strategy to obtain structurally and biochemically similar analog of H3K9me3, in which a tri-methyl-aminoethyl group was introduced to the thiol group of cysteine 9 in the context of H3K9C/C110A and H3K9C/S10A/C110A histone H3 samples.^22^ In brief, 10 mg lyophilized H3K9C/C110A or H3K9C/S10A/C110A H3 sample powder was solubilized in 980 μL solubilization buffer (1 M HepesNa, pH 7.4, 4 M guanidine hydrochloride, 10 mM D/L-methionine, 20 mM DTT) by incubation at 37 °C for 1 h. 100 mg bromocholine bromide (TCI) was then added to the solution to initiate the chemical reaction. After incubation at 50 °C for 2.5 h, 10 μL of 1 M DTT was added and the mixture was incubated for another 2.5 hs. By the end of the reaction, 50 μL 2-mercaptoethanol was added to quench extra unconsumed bromocholine bromide. Before octamer reconstitution, the MLA H3 proteins were exchanged to unfolding buffer (20 mM HepesNa, pH 7.4, 6 M guanidine hydrochloride, 10 mM DTT) using PD-10 desalting columns (GE Healthcare), and the tri-methylated lysine 9 analogs on H3K9C/C110A and H3K9C/S10A/S110A proteins were confirmed by western blot assays using monoclonal anti-Histone H3K9me3 antibody (Abcam).

### Reconstitution of 12× nucleosome chromatin

The respective histone octamers were reconstituted using the method of serial dialysis. Briefly, equal molar amounts of individual histones in unfolding buffer (20 mM HepesNa, pH 7.4, 6 M guanidine hydrochloride) were combined and dialyzed against refolding buffer (20 mM HepesNa, pH 7.4, 2 M NaCl). Octamers were further purified through a Superdex 200 10/300 GL column (GE Healthcare).

Reconstitution of 12× nucleosome chromatin was performed at 4 °C using the salt dialysis method as previously reported with minor modifications.^49^ DNA template harboring 12 × 177 bp tandem repeats of Widom 601 sequence and respective histone octamers were mixed together with the molar ratio of 1:12, and dialyzed over 18 h in HNE buffer (40 mM HepesNa, pH 7.4, 2 M NaCl, 0.5 mM EDTA, 1 mM DTT, 0.5 mM Benzamidine). The mixture was continuously diluted by slowly adding LNE buffer (40 mM HepesNa, pH 7.4, 350 mM NaCl, 0.5 mM EDTA, 1 mM DTT, 0.5 mM Benzamidine) to decrease the NaCl concentration from 2 M to 0.6 M. The reconstituted 12× nucleosome chromatin was further dialyzed against FNE buffer (20 mM HepesNa, pH 7.4, 50 mM NaCl) for 3 h before use.

### Negative stain and electron microscopy

Before grid preparation, all samples were co-dialyzed against the same buffer (20 mM HepesNa, pH 7.4, 50 mM NaCl) overnight, and then, 0.04 μM respective 12 × nucleosome chromatins were used for negative stain.

For negative staining, the copper grids supported by a thin layer of lacey carbon film (Beijing Zhongjingkeyi Technology, China) were glow-discharged. 4 mL of each sample at a concentration of ~ 0.04 μM were applied onto the grid for 1 min, and then the grid was washed in uranyl acetate (2% w/v) for 30 seconds before drying. Images were taken on an FEI Tecnai Spirit Bio TWIN microscope operating at 120 kV.

### Aurora B, HP1a, and HP1α purification

Human Aurora B kinase cDNA was cloned into a pET Duet (Novagen) with a His-MBP tag in N-terminus. HP1a and HP1α were cloned into a pRSFDuet vector (Novagen) with a 6 × His tag at the N-terminus. All proteins were expressed in *E. coli* BL21 (DE3). Cells were cultured to OD600 0.6 at 37 °C and induced at 18 °C with 0.5 mM IPTG overnight. Cells were ultrasonically broken down in the buffer (20 mM HepesNa 500 mM NaCl, pH 8.0), and then centrifuged at 20000 × g for 40 min. The supernatant was incubated with Ni resin followed by washing resin with washing buffer (40 mM imidazole, 20 mM HepesNa, pH 8.0, 500 mM NaCl). The proteins were eluted with elution buffer (400 mM imidazole, 20 mM HepesNa, pH 8.0, 500 mM NaCl). The proteins were further purified with ion exchange chromatography and size-exclusion chromatography.

### Aurora B treatment

Aurora B kinase, ATP (NEB, P0756S) and 10 × kinase buffer (CST, 9802S) were added to the nucleosome array. The final concentration of kinase was 5 µM and ATP was 50 µM. The mixture was incubated at 30 °C for 5 h. The reaction product was detected by the ph-H3S10 antibody (Abcam, ab5176). Phase separation experiment of H3K9me3S10ph nucleosome array was conducted using the following protocol.

### Phase separation experiments

ADCP1 Agenet domain 1-4 (1-355 aa) and Agenet 3-6 (201–517 aa) proteins were stored in 20 mM HepesNa, pH 7.4, 150 mM NaCl. A 1:1 molar ratio of Alex 488 (Invitrogen, A10254, dissolved in DMSO) was added into ADCP1 and the mixture was incubated at room temperature for 1 h. The excess dye was removed using MicroSpin G-50 column (GE Healthcare, 27533001). DAPI was added to native/K9me3/K9me3S10A 12× nucleosome array to label the DNA. Protein and nucleosome array were diluted to a proper concentration. Equal volume of nucleosome array and ADCP1 were mixed in a 384 well glass bottom plate (Cellvis, P384-1.5H-N). For Fig. 6a, the concentrations of three proteins were 30 μM. For Fig. 6c, the concentrations of both HP1A and HP1α were 25μ M (labelled with Alex 568 (Invitrogen, A20341, dissolved in DMSO) as described above), the concentration of H3K9me3 was 0.84 μM. For Fig. 6d, the concentration of ADCP1 was 15 μM, the concentration of H3K9me3 was 2.2 μM. The sample volume in each well was 5–10 μL. After 1 h incubation at room temperature, images were captured by Nikon A1RMP confocal microscope with 100 × oil objective. For FRAP analysis, mean fluorescence intensities from five regions of interests (ROIs) of time-lapse images were computed. ROI-1 was the bleached droplet, ROI-2 was a non-bleached droplet, ROI-3 was a background region. NIS-element software was used for data analysis.

### Data access

The sequencing data have been deposited into Gene Expression Omnibus (GEO) with the accession number GSE118063. The atomic coordinates and structure factors have been deposited into Protein Data Bank under accession codes 6IE4 for ADCP1-Agenet1/2-H3(1-15)K9me1, 6IE5 for ADCP1-Agenet3/4, 6IE6 for ADCP1-Agenet3/4-H3(1-15)K9me2, and 6IE7 for ADCP1-Agenet1/2-H3(1-15)K9me2.

## Electronic supplementary material

Supplementary information, Figure S1

Supplementary information, Figure S2

Supplementary information, Figure S3

Supplementary information, Figure S4

Supplementary information, Figure S5

Supplementary information, Figure S6

Supplementary information, Figure S7

Supplementary information, Table S1

Supplementary information, Table S2

Supplementary information, Table S3

Supplementary information, Table S4

Supplementary information, Table S6

Supplementary information, Table S5

Supplementary information, Table S7

Supplementary information, Figure S8

## Electronic supplementary material

**Supplementary information** accompanies for this paper at 10.1038/s41422-018-0104-9.
