# Supplementary material for: Plant HP1 protein ADCP1 links multivalent H3K9 methylation readout to heterochromatin formation
Source: Cell Res. 2018 Nov 13;29(1):54–66. doi: 10.1038/s41422-018-0104-9 (PMC6318295; doi:10.1038/s41422-018-0104-9)
Supplement: Supplementary file 1 — Supplementary information, Figure S1 [file 41422_2018_104_MOESM1_ESM.pdf]

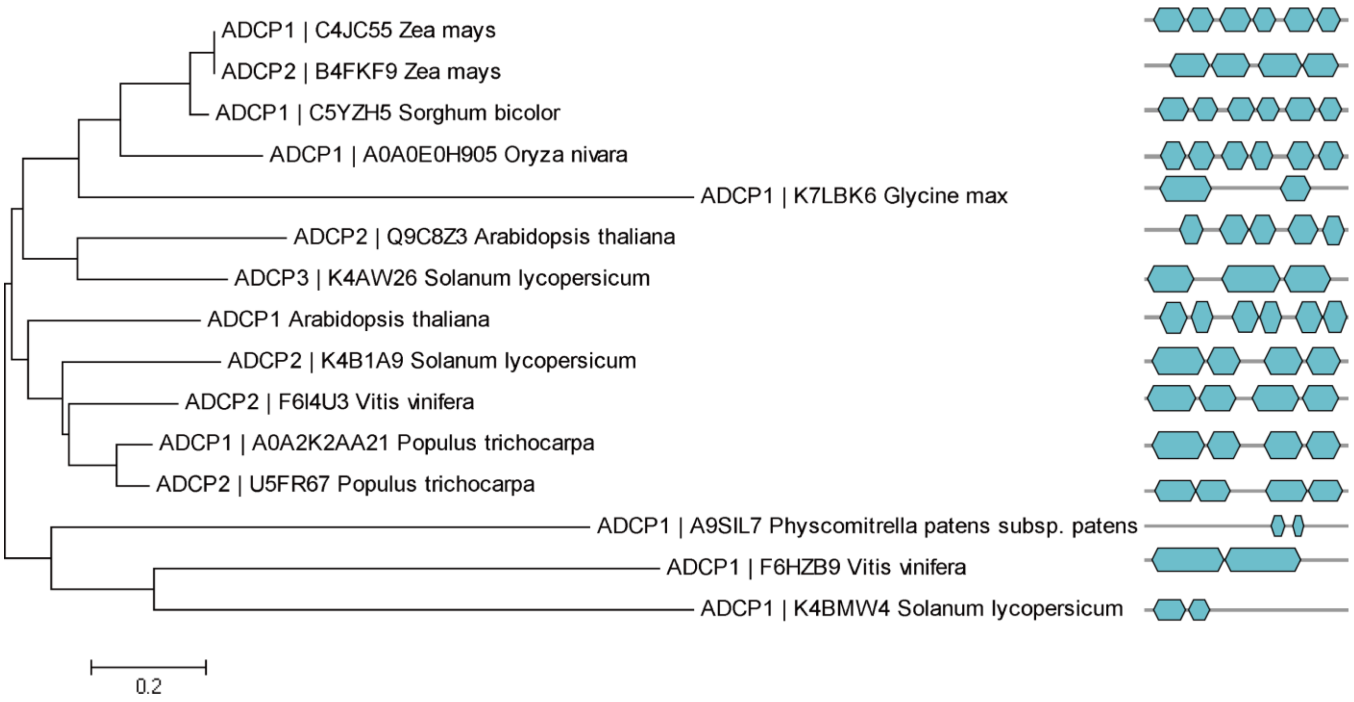

Figure S1 Phylogenetic tree of ADCP1 in different plant species. The phylogenetic tree of ADCP1 in different species, constructed by MEGA using Neighbor-Joining Tree method. The Agenet domain was shown as the colored hexagon.
