# Supplementary material for: Plant HP1 protein ADCP1 links multivalent H3K9 methylation readout to heterochromatin formation
Source: Cell Res. 2018 Nov 13;29(1):54–66. doi: 10.1038/s41422-018-0104-9 (PMC6318295; doi:10.1038/s41422-018-0104-9)
Supplement: Supplementary file 2 — Supplementary information, Figure S2 [file 41422_2018_104_MOESM2_ESM.pdf]

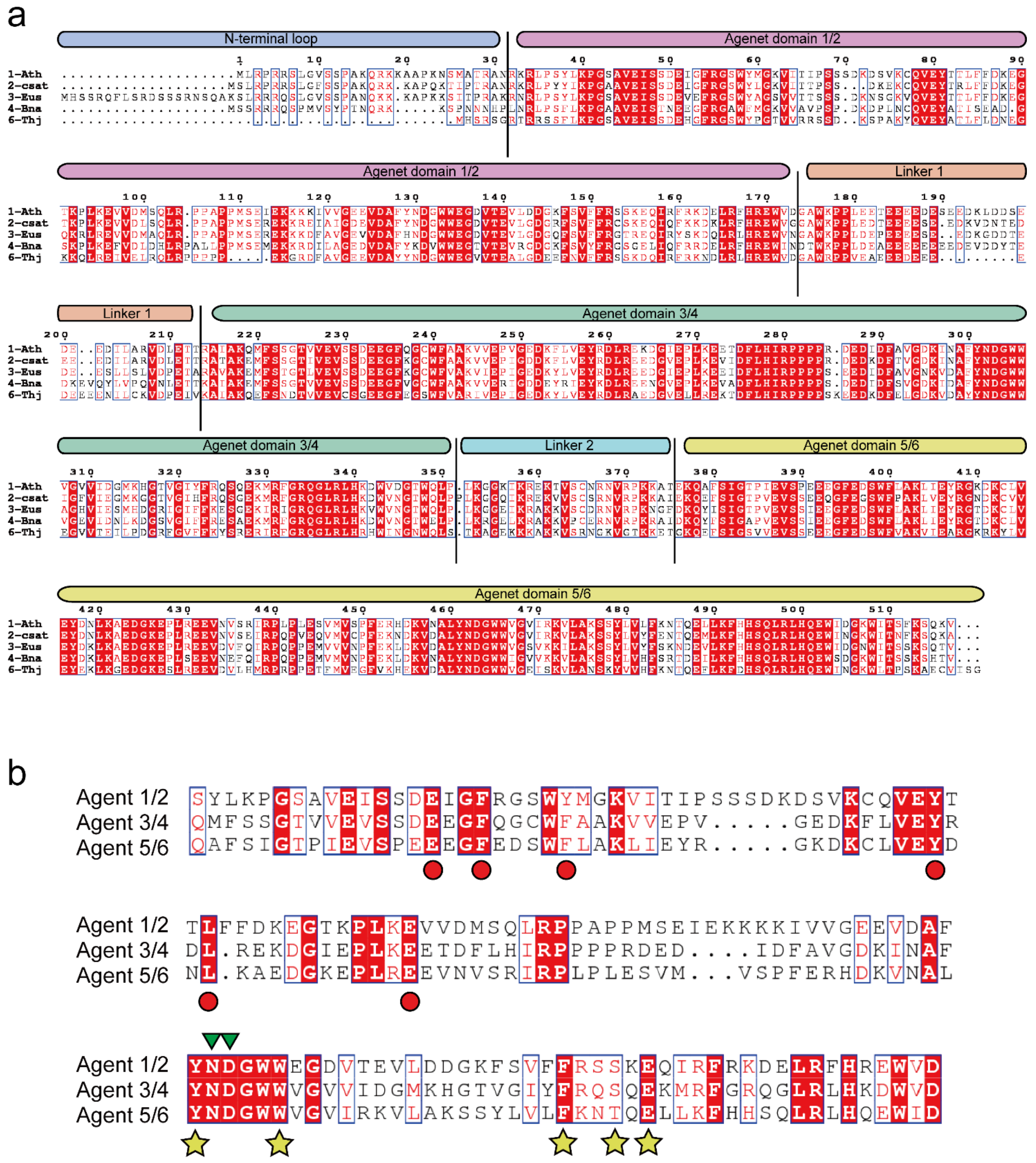

Figure S2 The sequence alignment of plant ADCP1. a The sequence alignment of ADCP1 in *Arabidopsis thaliana* (Ath), *Camelina sativa* (Csat), *Eutrema salsugineum* (Eus), *Brassica napus* (Bna) and *Tarenaya hassleriana* (Thj). **b** The sequence alignment of Agenet 1/2, Agenet 3/4 and Agenet 5/6 domains of *At*ADCP1. The residues involved in H3R2 recognition are labelled with green triangles; the residues involved in H3K4 recognition are labelled with red circles; the residues involved in H3K9me2 recognition are labelled with yellow asterisks.
