# Supplementary material for: Plant HP1 protein ADCP1 links multivalent H3K9 methylation readout to heterochromatin formation
Source: Cell Res. 2018 Nov 13;29(1):54–66. doi: 10.1038/s41422-018-0104-9 (PMC6318295; doi:10.1038/s41422-018-0104-9)
Supplement: Supplementary file 3 — Supplementary information, Figure S3 [file 41422_2018_104_MOESM3_ESM.pdf]

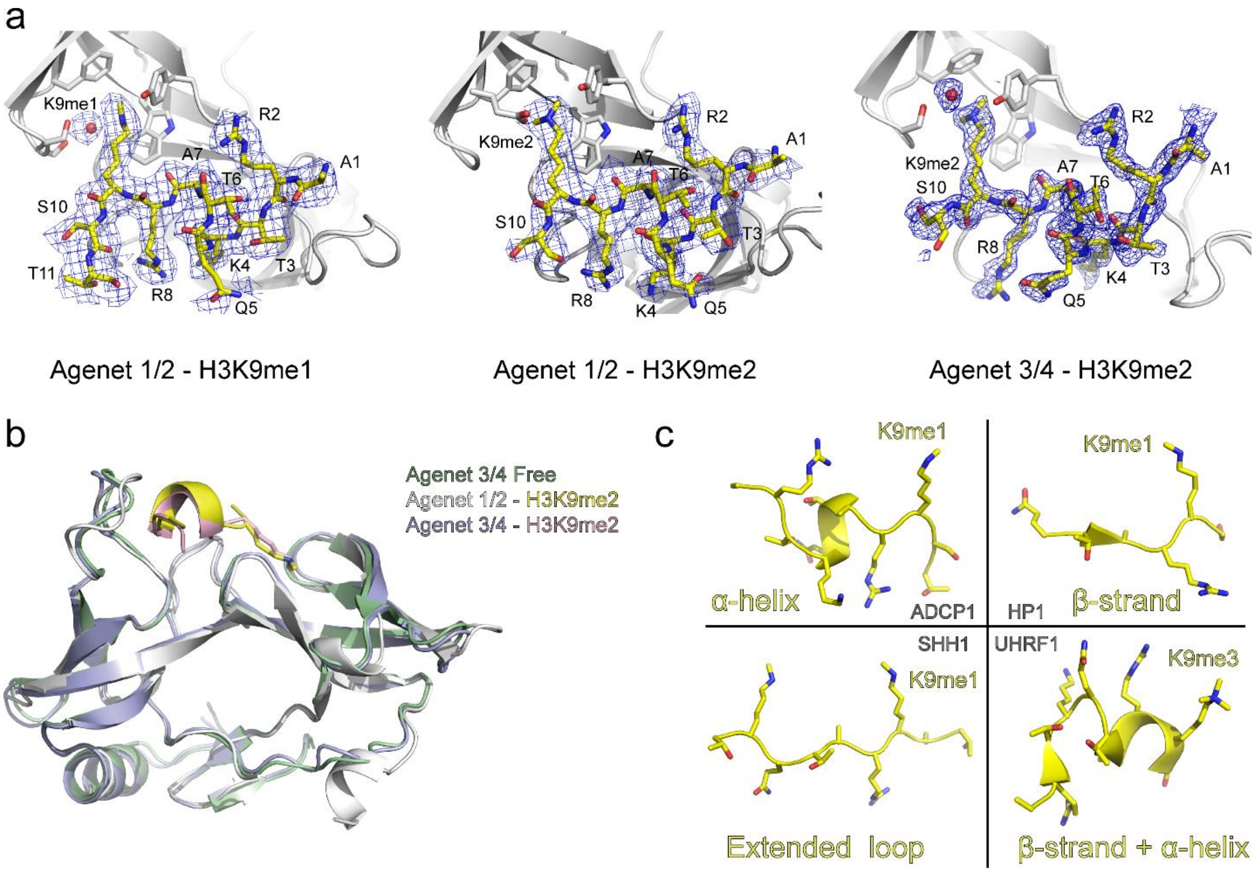

Figure S3 The crystal structures of ADCP1 in complex with H3K9me peptides. a The crystal structure of Agenet 1/2-H3K9me1, Agenet 1/2-H3K9me2 and Agenet 3/4-H3K9me2 complexes. Histone peptides are shown with a Fo-Fc omit map contoured at 2.0 σ. **b** The alignment of Agenet 1/2-H3K9me2, Agenet 3/4-free, Agenet 3/4-H3K9me2 structures. **c** The conformation of histone peptide in complex with histone H3K9me2/3 readers ADCP1, HP1, SHH1 and UHRF1. Only histone peptides are shown in the figure as cartoon model.
