# Supplementary material for: Plant HP1 protein ADCP1 links multivalent H3K9 methylation readout to heterochromatin formation
Source: Cell Res. 2018 Nov 13;29(1):54–66. doi: 10.1038/s41422-018-0104-9 (PMC6318295; doi:10.1038/s41422-018-0104-9)
Supplement: Supplementary file 4 — Supplementary information, Figure S4 [file 41422_2018_104_MOESM4_ESM.pdf]

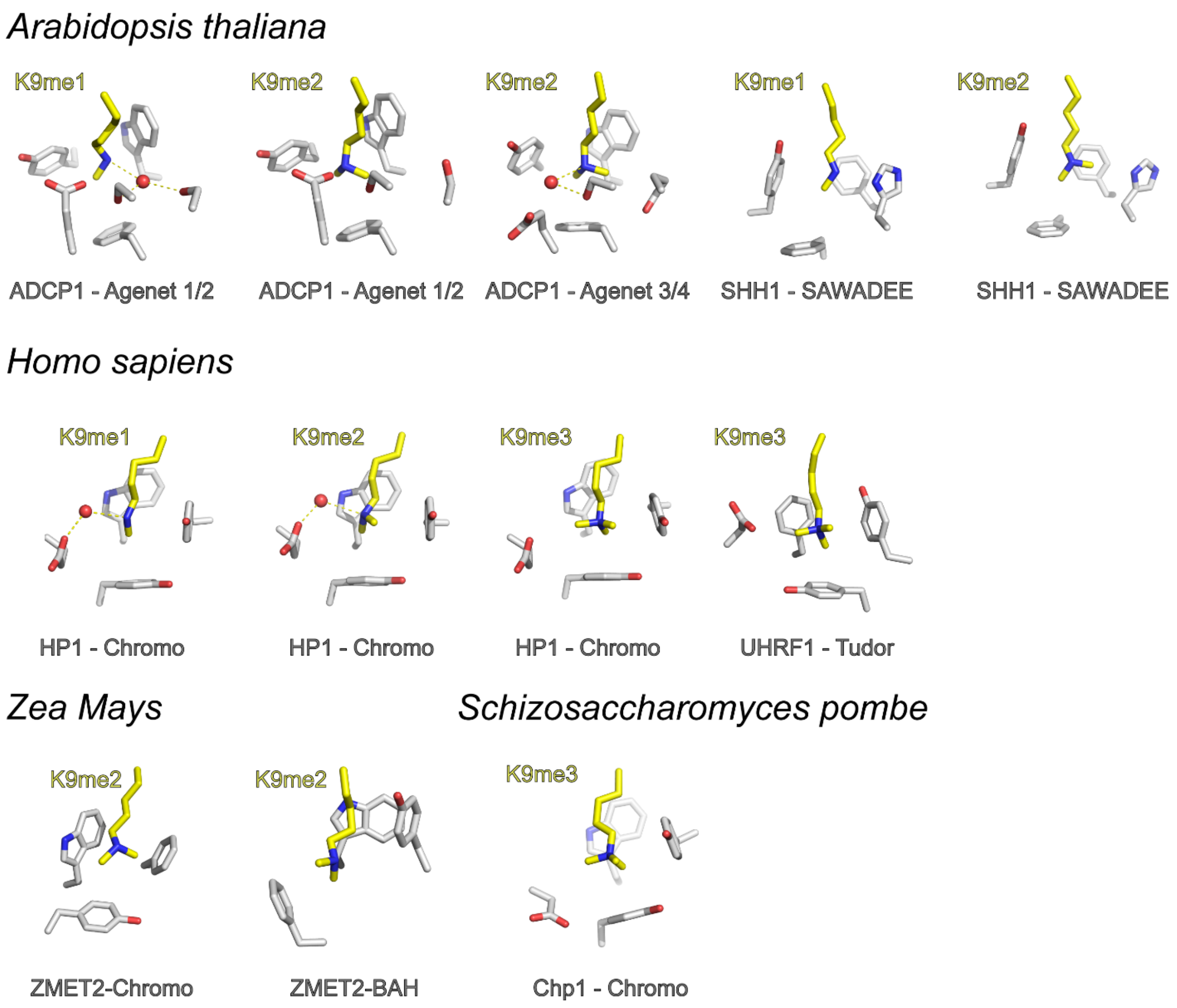

Figure S4 The comparison of H3K9me1/2/3 binding pockets in different readers. The binding pockets of H3K9me in histone readers in different species are shown here. Methylated H3K9 residue is shown in yellow; residues involved in H3K9me recognition are shown in white.
