# Supplementary material for: Plant HP1 protein ADCP1 links multivalent H3K9 methylation readout to heterochromatin formation
Source: Cell Res. 2018 Nov 13;29(1):54–66. doi: 10.1038/s41422-018-0104-9 (PMC6318295; doi:10.1038/s41422-018-0104-9)
Supplement: Supplementary file 5 — Supplementary information, Figure S5 [file 41422_2018_104_MOESM5_ESM.pdf]

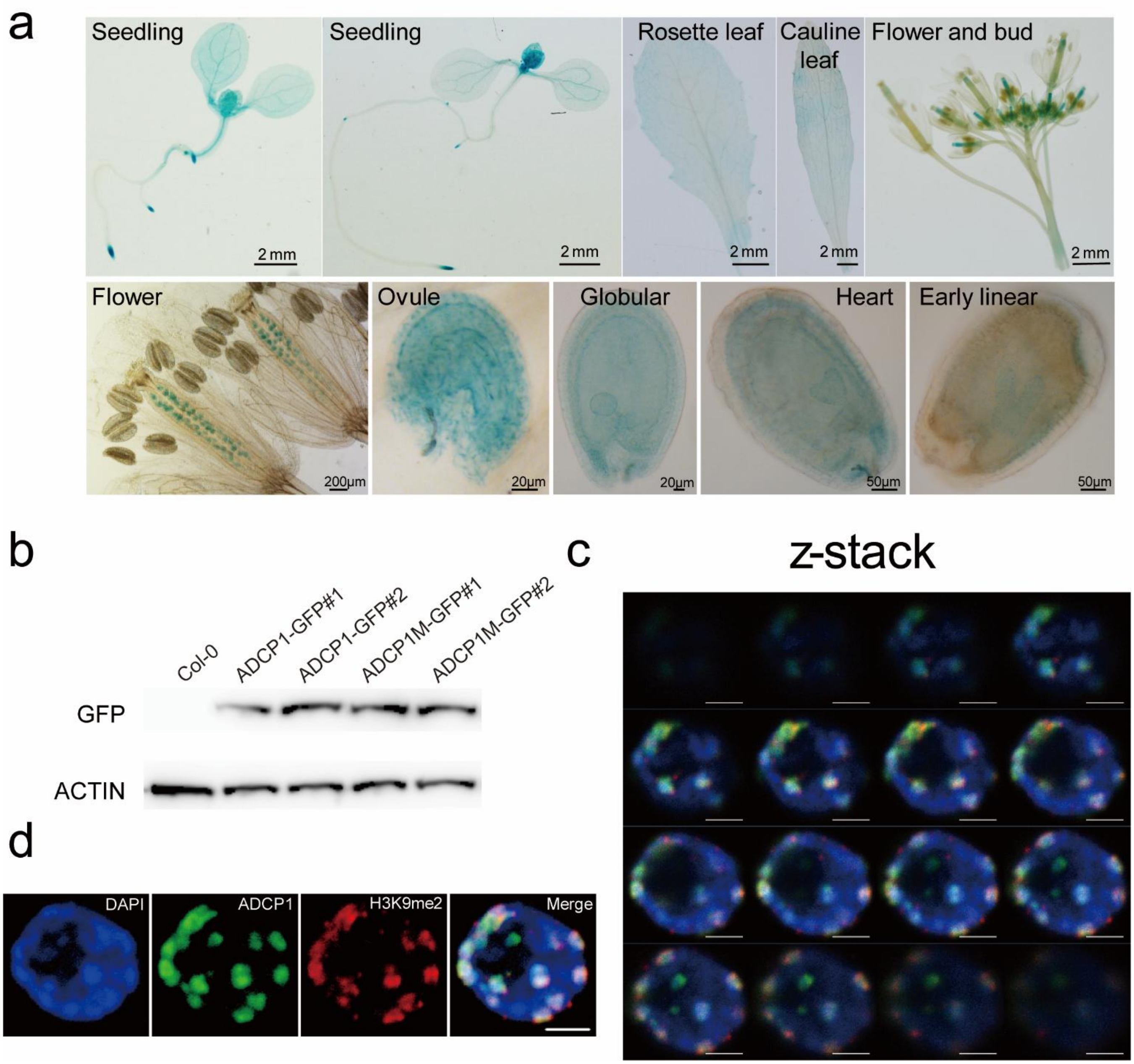

Figure S5 The expression pattern of ADCP1 in *Arabidopsis*. a GUS stainings of pADCP1:ADCP1:GUS transgenic plants including 7 day-old seedlings, rosette leaf, cauline leaf, flower, buds, ovule, and seeds at globular stage, heart stage and linear stage were shown. **b** Western Blot of ADCP1/ADCP1M-GFP complementary transgenic plants in *adcp1-1* with anti-GFP antibody (ADCP1-GFP is approximately 86kDa in molecular weight), Col-0 was used as a negative control. ACTIN was used as a loading control. **c** The Gallery of the z-stack scan shown in Fig. 3a. Bar= 2 μm. **d** The 3D Reconstruction of Fig. 3a. Bar= 2 μm.
