# Supplementary material for: Plant HP1 protein ADCP1 links multivalent H3K9 methylation readout to heterochromatin formation
Source: Cell Res. 2018 Nov 13;29(1):54–66. doi: 10.1038/s41422-018-0104-9 (PMC6318295; doi:10.1038/s41422-018-0104-9)
Supplement: Supplementary file 6 — Supplementary information, Figure S6 [file 41422_2018_104_MOESM6_ESM.pdf]

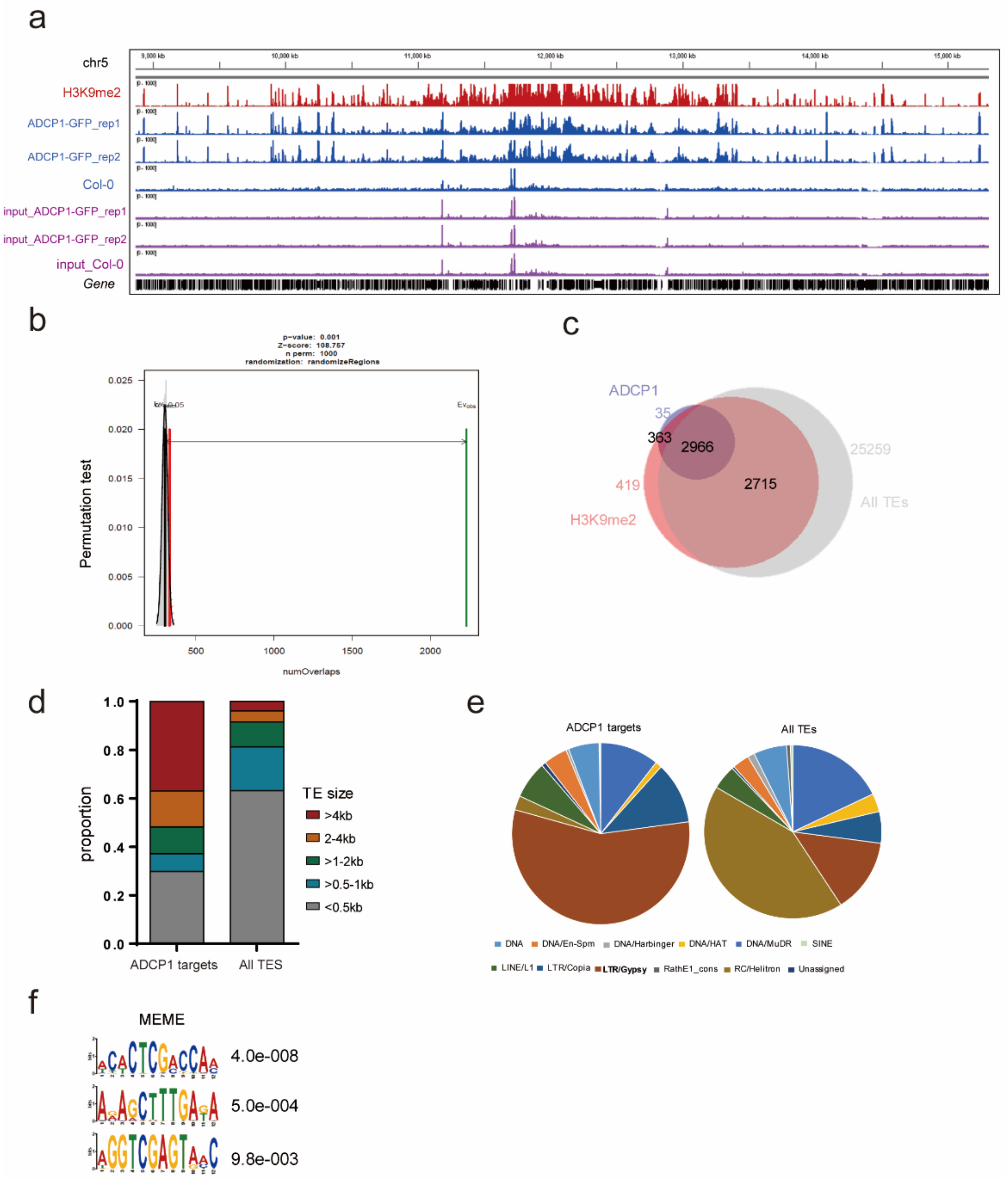

Figure S6 The targets of ADCP1 are the subset of H3K9me2 marked regions. a IGV view of ADCP1 and H3K9me2 signals in more than 6,000 kb long region on chromosome 5. The data are shown

as RPKM. **b** 1000 times permutation test of the association of H3K9me2 and ADCP1 peaks using RegioneR. The number of overlaps of the randomized regions with H3K9me2 is in grey, clustering around the black bar that represents the mean and the number of overlaps of ADCP1 and H3K9me2 is in green, which is much larger than expected. The red line denotes the significance limit. **c** Venn diagram between ADCP1, H3K9me2 targets and all TEs. 2966 out of 3364 ADCP1-bound loci belong to H3K9me2-marked TEs. The exclusive number of loci among ADCP1, H3K9m2 and all TEs are indicated outside the circles. ADCP1 targets are listed at Supplementary Information, Table S4. **d** The proportion of TEs in different lengths including ADCP1-targed TEs and all TEs. **e** The classification of ADCP1-targeted TEs and all TEs as a control. **f** The motifs of ADCP1 targets found by MEME, other motifs are shown at Table S3 found by homer.
