# Supplementary material for: Plant HP1 protein ADCP1 links multivalent H3K9 methylation readout to heterochromatin formation
Source: Cell Res. 2018 Nov 13;29(1):54–66. doi: 10.1038/s41422-018-0104-9 (PMC6318295; doi:10.1038/s41422-018-0104-9)
Supplement: Supplementary file 7 — Supplementary information, Figure S7 [file 41422_2018_104_MOESM7_ESM.pdf]

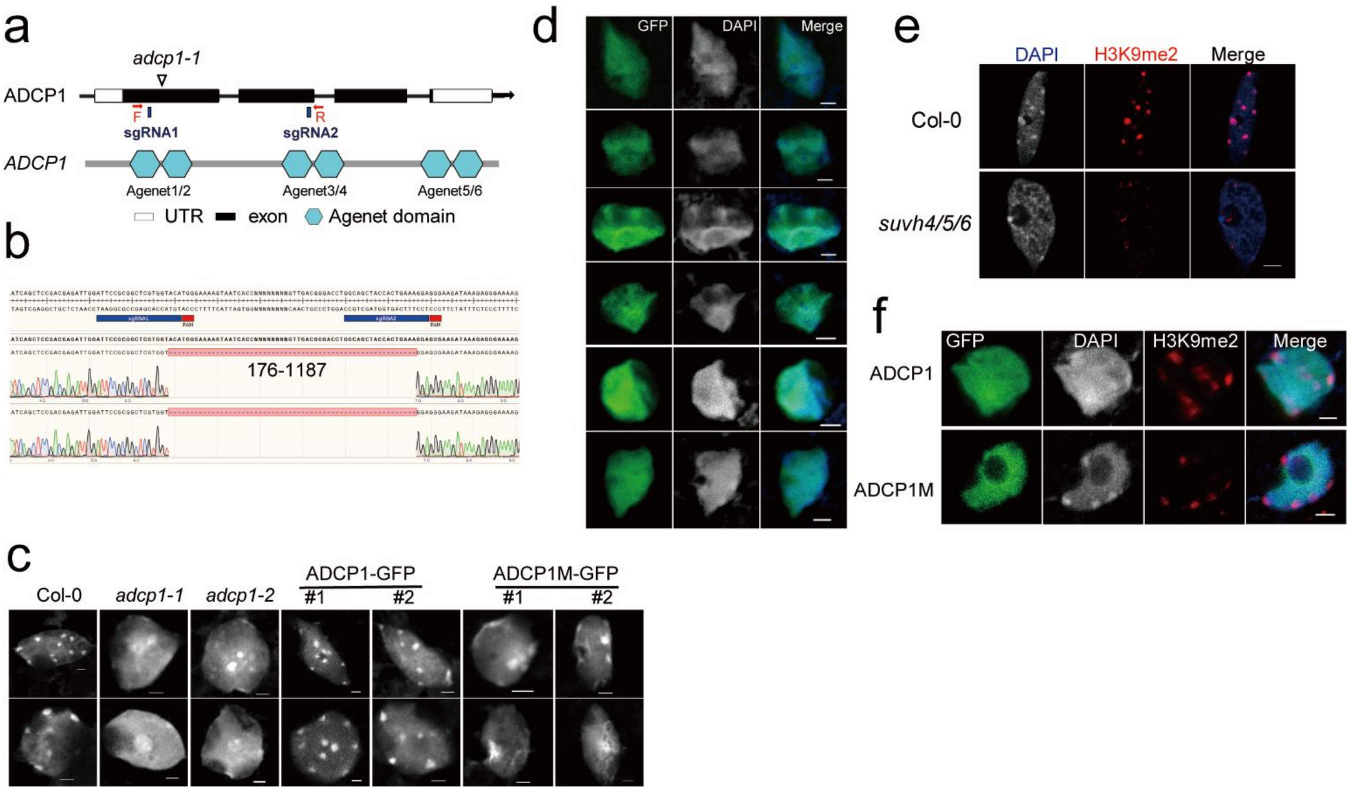

Figure S7 ADCP1 is required for nuclei heterochromatic chromocenter formation. a The gene and protein structure of ADCP1. T-DNA mutant of *adcp1-1* was inserted in the first exon. Two sgRNA that generated the deletion mutant *adcp1-2* were indicated. **b** The sequencing data of F and R (red arrows shown in panel a) PCR products in two individual *adcp1-2* deletion plants. The sgRNAs are showed in panel a. *adcp1-2* carries the deletion from 178bp to1187bp in the genomic sequence (counted from ATG) of ADCP1 and causes premature termination before Agenet 5/6. **c** Representative nuclear condensation status stained with DAPI. Bar= 2 μm. **d** Different nuclei with transient overexpressed ADCP1 in Col-0 protoplasts. Bar= 2 μm. **e** H3K9me2 immunostaining in the interphase of *suvh456* nuclei. Bar= 2 μm. **f** H3K9me2 immunostaining in transient transformed Col-0 protoplasts with ADCP1 or ADCP1M after 14-hour expression. Bar= 2 μm.
