# Supplementary material for: Plant HP1 protein ADCP1 links multivalent H3K9 methylation readout to heterochromatin formation
Source: Cell Res. 2018 Nov 13;29(1):54–66. doi: 10.1038/s41422-018-0104-9 (PMC6318295; doi:10.1038/s41422-018-0104-9)
Supplement: Supplementary file 8 — Supplementary information, Table S1 [file 41422_2018_104_MOESM8_ESM.pdf]

Table S1. Data collection and refinement statistics of crystallography.

	Agenet 1/2 -H3(1-15)K9me1	Agenet 1/2 -H3(1-15)K9me2	Agenet 3/4 –Free	Agenet 3/4 -H3(1-15)K9me2
Data collection	Native	Native	Se-Met	Native
Space group	$P2_12_12_1$	$P2_1$	$P6_522$	$P6_522$
Cell dimensions				
a, b, c (Å)	42.5, 67.5, 116.7	41.5, 111.3, 68.1	56.2, 56.2, 188.3	56.9, 56.9, 210.0
α, β, γ (°)	90, 90, 90	90, 90.6, 90	90, 90, 120	90, 90, 120
Resolution (Å)	50-2.7 (2.75-2.70)*	50-2.7 (2.75-2.70)	50-2.3 (2. 34-2.30)*	50-1.7 (1.73-1.70)
R_{sym} or R_{merge}	0.095 (0.479)	0.066 (0.295)	0.204 (0.662)	0.066 (0.724)
I / σI	13.2 (2.4)	14.9 (2.5)	16.5 (5.0)	40.7 (1.9)
Completeness (%)	99.9 (99.8)	89.1 (91.9)	99.8 (100.0)	95.7 (97.0)
Redundancy	6.2 (5.7)	2.5 (2.5)	13.7 (13.4)	5.8 (6.1)
Refinement				
Resolution (Å)	34.4-2.7	33.9-1.7	38.5-2.3	32.0-1.7
No. reflections	9752	15155	8514	6352
R_{work} / R_{free}	0.202/0.269	0.190/0.256	0.184/0.234	0.186/0.232
No. atoms				
Protein	2221	4658	1205	1298
Peptide	174	224	/	58
Water	38	48	60	168
B-factors ($Å^2$)				
Protein	46.9	44.8	17.9	47.4
Peptide	46.2	40.5	/	49.8
Water	39.7	36.4	19.1	53.9
R.m.s. deviations				
Bond lengths (Å)	0.009	0.009	0.008	0.007
Bond angles (°)	1.136	1.260	1.160	1.030

* Values in parentheses are for highest-resolution shell.
