# Supplementary material for: Plant HP1 protein ADCP1 links multivalent H3K9 methylation readout to heterochromatin formation
Source: Cell Res. 2018 Nov 13;29(1):54–66. doi: 10.1038/s41422-018-0104-9 (PMC6318295; doi:10.1038/s41422-018-0104-9)
Supplement: Supplementary file 9 — Supplementary information, Table S2 [file 41422_2018_104_MOESM9_ESM.pdf]

Table S2. Thermodynamic fitting parameters from isothermal titration calorimetry (ITC)

Protein	Peptide	Ka (10^5*M^{-1})	ΔH (kcal/mol)	ΔS (cal/mol/deg)	N
Agenet 1-2	$H3_{(1-15)}$K9me1	1.41±0.09	-9.55±0.09	-9.60	1.18±0.87
	$H3_{(1-15)}$K9me2	3.33±0.01	-10.02±0.06	-10.20	1.01±0.08
	$H3_{(1-15)}$K9me3	1.55±0.03	-10.45±0.03	-12.50	1.04±0.02
	$H3_{(1-15)}$K4me3	0.19±0.03	-2.10±0.18	12.30	1.12±0.07
	$H3_{(1-15)}$K9me2S10ph	0.41±0.04	-9.8±0.33	-11.90	1.04±0.03
Agenet 3-4	$H3_{(1-15)}$K9me1	0.41±0.07	-4.84±0.18	4.89	1.08±0.01
	$H3_{(1-15)}$K9me2	0.47±0.05	-5.18±0.15	4.04	1.09±0.02
	$H3_{(1-15)}$K9me3	0.28±0.03	-4.00±0.14	6.97	1.13±0.02
	$H3_{(1-15)}$K4me3	0.24±0.04	-3.55±0.28	8.17	0.971±0.05
	$H3_{(1-15)}$K9me2S10ph	0.10±0.01	-5.43±0.30	0.02	1.00 (fixed)
Agenet 5-6	$H3_{(1-15)}$K9me1	1.26±0.20	-6.94±0.31	0.06	1.03±0.03
	$H3_{(1-15)}$K9me2	1.73±0.19	-6.14±0.17	3.37	1.08±0.02
	$H3_{(1-15)}$K9me3	0.77±0.07	-6.33±0.21	1.13	0.955±0.02
	$H3_{(1-15)}$K9me2S10ph	0.39±0.05	-7.69±0.53	-4.77	0.996±0.05
