# Supplementary material for: Plant HP1 protein ADCP1 links multivalent H3K9 methylation readout to heterochromatin formation
Source: Cell Res. 2018 Nov 13;29(1):54–66. doi: 10.1038/s41422-018-0104-9 (PMC6318295; doi:10.1038/s41422-018-0104-9)
Supplement: Supplementary file 10 — Supplementary information, Table S3 [file 41422_2018_104_MOESM10_ESM.pdf]

Table S3. Primers

Usage	Name	Sequence
genotyping	*adcp1-1*_LP	TGCATCTTCTGTTCATGTTGC
	*adcp1-1*_RP	CTCCTCGTCTTCTGAGTCGTC
	adcp1-2_F	TTCCATGGCTACGAGAGCGAAT
	adcp1-2_R	CTTTCTTGGGTCTCACGTTCCG
qPCR	UBC_qPCR_F	CTGCGACTCAGGGAATCTTCTAA
	UBC_qPCR_R	TTGTGCCATTGAATTGAACCC
	qGAPDH-F	TTGGTGACAACAGGTCAAGCA
	qGAPDH-R	AAACTTGTCGCTCAATGCAATC
	AT3TE45385-F	GGCAACCTAAGATGCTTGAAGCT
	AT3TE45385-R	GGCCATCTGAGAATTCTACCACC
	AT5TE34980-F	CACCCTCCAATTACATTTGCCAC
	AT5TE34980-R	GAGGTGACATTGAAACATGGGC
	AT3TE76225-F	CTCCACCAACCTTCAGAGATGTG
	AT3TE76225-R	CGGTTGCCGGAAGTGTAGTTG
	AT3TE45390-F1	CTCTCCCAGGCACGAGTTTC
	AT3TE45390-R1	TGAGACGCCGGAATAACGGG
	AT3TE68090-F	TCTGCTGATAATCCTGGAGCCG
	AT3TE68090-R	GAAGAGCTTGAATCGTCGTCCAG
	AT5TE61735_F	CAACGTCTCAGCTCCTCAAAGTG
	AT5TE61735_R	GGCTATCACGTCCTGTATCTGACTC
	qPCR-TSI-R	TCACTTGTGAGTGTTCGTGAGGTC
	qPCR-TSI-F	ATCCAGTCCGAAGAACGCGAACTA
	qPCR-CACTA-R	AACTTACATGTTTGCGGGCACGAG
	qPCR-CACTA-F	TGTGTGGAAGGGTCTTGTGGACTT
	qPCR-Ta3-R	ACGCCCTTTACCTTGACCTCCTTT
	qPCR-Ta3-F	AAGAGAGCTGGCAGAAGCAGTTGA
Vector Construction		
CRISPR	sgRNA1	ATATATGGTCTCGATTGTTCCGCGGCTCGTGGTACAGTTTTAGAG CTAGAAATAGC
	sgRNA2	ATTATTGGTCTCGAAACTCAATCCATTCCTGATGTACAATCTCTT AGTCGACTCTAC
35S:ADCP1 : GFP	ADCP1-F(PUC)	CTCGGTACCCTCGAGATGTTACGTCCTCGTCGC
	ADCP1-R(PUC)	CTTGCTCACTTCGAACACTTTCTGGCTTTTGAAGC
	pUC19-GFP-RP	TTCGAAGTGAGCAAGGGCGAG
	pUC19-LP	CTCGAGGGTACCGAGCTCGT
ADCP1 complement ation	ADCP1-pro-F	ATTAGGCACCCCAGGCTTCAGTTGCAGCATAAGCT
	ACDP1-ter-R	CATTCGCCATTCAGGAGCTTGTCTCCTATGGAACCG
	pBar-LP	CCTGAATGGCGAATGCTAGAGC
	pBar-RP	CCTGGGGTGCCTAATGAGTGT
	pBar-ADCP1-LB(GUS)	CAGGGAGGCAAACAATAAATGGGCTGGATAACTCG
	pBar-ADCP1-RB(GUS)	TTCTACAGGACGTAACACCTTCTGAGACTGAAATC
	pBar-ADCP1-RB(eGFP)	CTCGCCCTTGCTCACCACCTTCTGAGACTGAAATC
	pBar-ADCP1-LB(eGFP)	GACGAGCTGTACAAGTAAATGGGCTGGATAACTCG
	eGFP-F	GTGAGCAAGGGCGAGGAG
	eGFP-R	CTTGTACAGCTCGTCCATGC
	GUS-F	TTACGTCCTGTAGAAACCCC
	GUS-R	TTGTTTGCCTCCCTGCTGC
