# Supplementary material for: Plant HP1 protein ADCP1 links multivalent H3K9 methylation readout to heterochromatin formation
Source: Cell Res. 2018 Nov 13;29(1):54–66. doi: 10.1038/s41422-018-0104-9 (PMC6318295; doi:10.1038/s41422-018-0104-9)
Supplement: Supplementary file 11 — Supplementary information, Table S4 [file 41422_2018_104_MOESM11_ESM.pdf]

**Table S4. *De novo* motifs of ADCP1 ChIP-seq found by homer**

| Rank | Motif | P-value | log P-pvalue | % of Targets | % of Background |
|---|---|---|---|---|---|
| 1 | 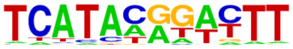 | 1e-116 | -2.686e+02 | 6.78% | 0.49% |
| 2 | 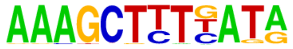 | 1e-111 | -2.576e+02 | 6.43% | 0.45% |
| 3 | 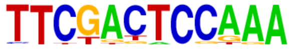 | 1e-91 | -2.106e+02 | 3.86% | 0.14% |
| 4 | 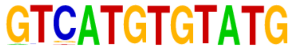 | 1e-79 | -1.835e+02 | 4.30% | 0.27% |
| 5 | 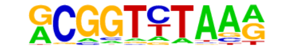 | 1e-76 | -1.752e+02 | 3.50% | 0.16% |
| 6 | 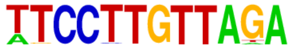 | 1e-75 | -1.742e+02 | 4.39% | 0.32% |
| 7 | 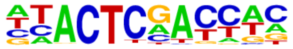 | 1e-71 | -1.654e+02 | 15.52% | 5.23% |
| 8 | 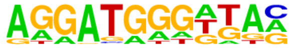 | 1e-68 | -1.584e+02 | 4.04% | 0.30% |
| 9 | 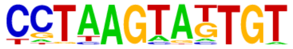 | 1e-68 | -1.571e+02 | 3.64% | 0.22% |
| 10 | 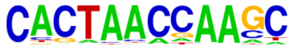 | 1e-65 | -1.499e+02 | 4.35% | 0.40% |
| 11 | 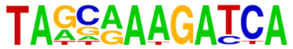 | 1e-63 | -1.453e+02 | 2.22% | 0.05% |
| 12 | 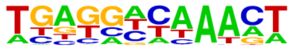 | 1e-63 | -1.452e+02 | 3.06% | 0.15% |
| 13 | 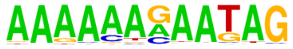 | 1e-62 | -1.430e+02 | 4.17% | 0.39% |
| 14 | 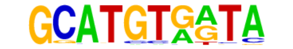 | 1e-59 | -1.368e+02 | 3.59% | 0.28% |
| 15 | 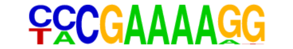 | 1e-58 | -1.340e+02 | 2.88% | 0.15% |
| 16 | 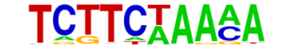 | 1e-58 | -1.338e+02 | 4.88% | 0.64% |
| 17 | 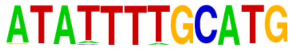 | 1e-57 | -1.331e+02 | 2.75% | 0.13% |
| 18 | 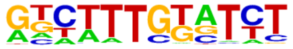 | 1e-54 | -1.250e+02 | 4.17% | 0.48% |
| 19 | 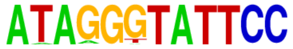 | 1e-54 | -1.245e+02 | 2.04% | 0.06% |
| 20 | 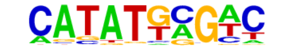 | 1e-53 | -1.227e+02 | 3.10% | 0.23% |
| 21 | 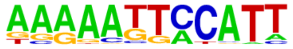 | 1e-53 | -1.227e+02 | 1.91% | 0.05% |
| 22 | 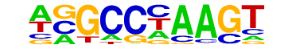 | 1e-52 | -1.204e+02 | 9.14% | 2.59% |
| 23 | 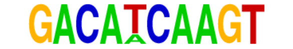 | 1e-50 | -1.174e+02 | 3.28% | 0.29% |

| | | | | | |
|---|---|---|---|---|---|
| 24 | 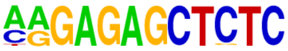 | 1e-50 | -1.153e+02 | 1.77% | 0.04% |
| 25 | 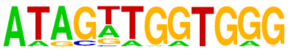 | 1e-47 | -1.098e+02 | 2.26% | 0.11% |
| 26 | 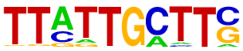 | 1e-46 | -1.082e+02 | 3.86% | 0.49% |
| 27 | 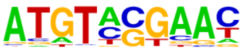 | 1e-43 | -9.918e+01 | 2.53% | 0.19% |
| 28 | 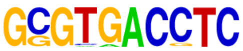 | 1e-41 | -9.605e+01 | 2.88% | 0.28% |
| 29 | 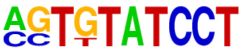 | 1e-39 | -9.116e+01 | 3.46% | 0.48% |
| 30 | 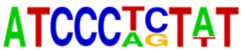 | 1e-38 | -8.876e+01 | 2.26% | 0.17% |
| 31 | 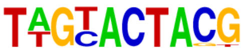 | 1e-38 | -8.805e+01 | 2.39% | 0.20% |
| 32 | 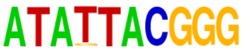 | 1e-33 | -7.819e+01 | 1.29% | 0.04% |
| 33 | 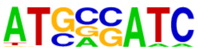 | 1e-33 | -7.630e+01 | 9.80% | 3.95% |
| 34 | 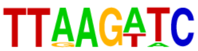 | 1e-30 | -6.981e+01 | 9.05% | 3.66% |
| 35 | 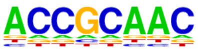 | 1e-26 | -6.033e+01 | 3.41% | 0.75% |
| 36 | 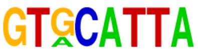 | 1e-23 | -5.326e+01 | 3.33% | 0.80% |
| 37 | 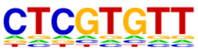 | 1e-20 | -4.639e+01 | 2.71% | 0.62% |
| 38 | 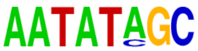 | 1e-18 | -4.339e+01 | 2.88% | 0.74% |
