# Supplementary material for: Plant HP1 protein ADCP1 links multivalent H3K9 methylation readout to heterochromatin formation
Source: Cell Res. 2018 Nov 13;29(1):54–66. doi: 10.1038/s41422-018-0104-9 (PMC6318295; doi:10.1038/s41422-018-0104-9)
Supplement: Supplementary file 12 — Supplementary information, Table S6 [file 41422_2018_104_MOESM12_ESM.pdf]

Table S6: The list of de-repressed TEs in *adcp1-1*

ID	Related TE_genes	log_2FoldChange	Family	SuperFamily	p_value
AT1TE22850	AT1G20390	1.00	ATLANTYS1	LTR/Gypsy	4.1E-05
AT1TE30555	AT1G27285	1.34	AT9TSD1	DNA/MuDR	0.00039
AT1TE93275	AT1G75945	2.40	HELITRON1	RC/Helitron	1.5E-14
AT2TE01550	AT2G01840	∞	ATLINE1_5	LINE/L1	0.00508
AT2TE08225	AT2G05040	4.10	ATGP3	LTR/Gypsy	0.00031
AT2TE28280	AT2G15940	4.81	TAG2	DNA/HAT	0.00024
AT2TE29460	AT2G16680	∞	TA11	LINE/L1	0.001
AT2TE37050	AT2G20460	4.58	ATCOPIA72	LTR/Copia	0.00011
AT3TE45385	AT3G28915	∞	ATLINE1_6	LINE/L1	0.00005
AT3TE60310	AT3G42658	1.93	SADHU	Unassigned	8.1E-05
AT3TE64435	AT3G44215	∞	ATCOPIA11	LTR/Copia	0.03254
AT3TE68090	AT3G45775	∞	ATCOPIA81	LTR/Copia	0.00005
AT3TE76225	AT3G50625	∞	ATCOPIA52	LTR/Copia	0.02257
AT3TE90530	AT3G60164	∞	ATCOPIA23	LTR/Copia	0.01599
AT4TE10600	AT4G04560	4.64	ATCOPIA57	LTR/Copia	0.04976
AT4TE67490	AT4G28900	1.28	ATCOPIA46	LTR/Copia	0.00025
AT5TE00480	AT5G01335	2.27	Unassigned	Unassigned	0.01965
AT5TE08220	AT5G07215	5.10	Unassigned	Unassigned	0.00012
AT5TE22500	AT5G18633	2.68	TA11	LINE/L1	0.03278
AT5TE23185	AT5G19097	4.98	ATCOPIA89	LTR/Copia	3.7E-21
AT5TE34980	AT5G27345	3.34		Unassigned	2.4E-05
AT5TE48605	AT5G35425	∞	VANDAL9	DNA/MuDR	0.02347
AT5TE50260	AT5G35935	5.04	ATCOPIA18A	LTR/Copia	7.7E-29
AT5TE56645	AT5G39155	4.09	ATCOPIA83	LTR/Copia	0.01224
AT5TE56665	AT5G39185	∞	ATCOPIA83	LTR/Copia	0.02056
AT5TE64385	AT5G44255	2.67	ATGP3	LTR/Gypsy	0.03785
AT5TE69650	AT5TE69650	∞		Unassigned	0.01296
AT2TE29465		∞	TA11	LINE/L1	0.001
AT3TE45390		∞	ATLINE1_4	LINE/L1	0.00005
AT3TE70640		∞	ATHATN3	DNA/HAT	0.1387
AT4TE10605		∞	ATCOPIA42	LTR/Copia	1.2E-05
AT5TE66495		∞	VANDAL3	DNA/MuDR	0.00005
AT2TE28285		4.81	ATHAT1	DNA/HAT	0.00024
AT2TE10570		3.52	ATHILA4C	LTR/Gypsy	0.04074
AT2TE10575		3.52	ATHILA4C	LTR/Gypsy	0.04074
AT4TE34015		2.97	VANDAL1	DNA/MuDR	0.00831
AT5TE64390		2.14	VANDAL16	DNA/MuDR	0.043
AT2TE77005		1.97	ATGP9B	LTR/Gypsy	0.00005
AT1TE12295		1.60	ATCOPIA78	LTR/Copia	0.00005
AT3TE51150		1.45	ATENSPM11	DNA/En-Spm	0.00005
AT2TE28290		1.37	ATHAT1	DNA/HAT	0.00024
AT2TE77015		1.14	ATGP9B	LTR/Gypsy	0.00005
AT2TE09335		1.12	BRODYAGA1	DNA/MuDR	0.00005
AT2TE09340		1.10	BRODYAGA1A	DNA/MuDR	0.00073
AT1TE98090		1.03	ATREP15	RC/Helitron	0.00005
