# Supplementary material for: Plant HP1 protein ADCP1 links multivalent H3K9 methylation readout to heterochromatin formation
Source: Cell Res. 2018 Nov 13;29(1):54–66. doi: 10.1038/s41422-018-0104-9 (PMC6318295; doi:10.1038/s41422-018-0104-9)
Supplement: Supplementary file 15 — Supplementary information, Figure S8 [file 41422_2018_104_MOESM15_ESM.pdf]

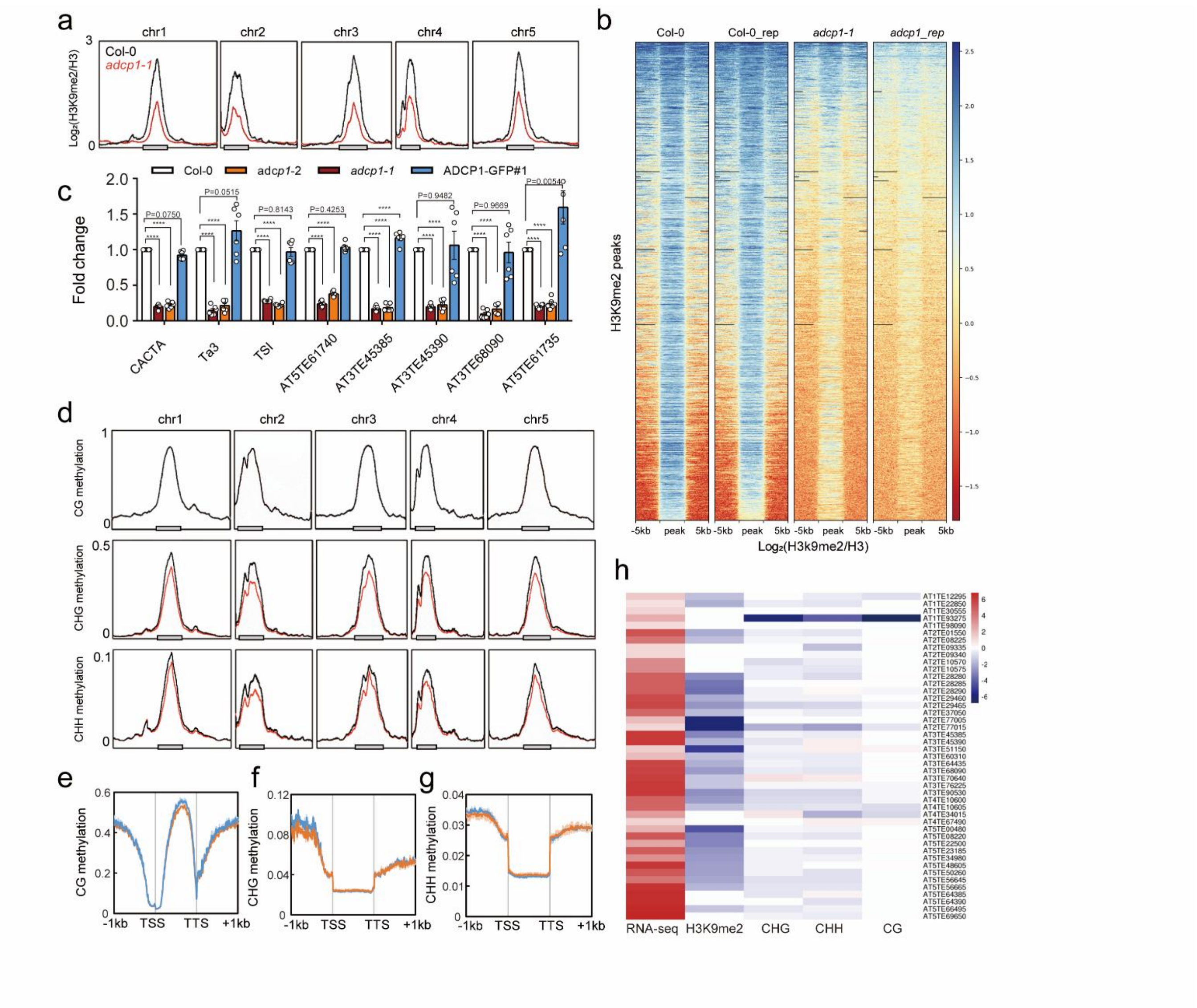

Figure S8 ADCP1 is required for the maintenance of H3K9me2 and CHG/CHH methylation, as well as TE silencing. a The distribution of H3K9me2 along five chromosomes. The gray boxes indicate the pericentromeric regions. The data show the mean of two biological replicates and plotted following smoothed using LOESS method by GraphPad Prism. **b** Heatmap of H3K9me2 peaks. **c** H3K9me2 ChIP-qPCR validation. The data were shown as *adcp1* (H3K9me2/H3)/Col-0(H3K9me2/H3). Error bar indicates SD. The circles represent the original data. One-way ANOVA is used for the statistical analysis, ****P<0.0001. **d** The distribution of CG methylation, CHG methylation and CHH methylation along chromosomes. The data show the mean of two biological replicates and plotted following smoothed using LOESS method by GraphPad Prism. **e-g** Metaplot of CG methylation (**e**), CHG (**f**) and CHH (**g**) in protein coding genes. The shadow means SD of two biological replicates. **h** Heatmap of H3K9me2 and DNA methylation at up-regulated TEs. The data shown as $\log_2$ (*adcp1*/Col-0).
